# Supplementary material for: Bottleneck and enabler evaluation of avian influenza health event — Guatemala, January-February 2023
Source: PLOS Glob Public Health. 2025 Nov 6;5(11):e0005443. doi: 10.1371/journal.pgph.0005443 (PMC12591453; doi:10.1371/journal.pgph.0005443)
Supplement: S1 Text — (DOCX) [file pgph.0005443.s001.docx]

**S1 Text: Interview Questions**

**Spanish Version**

1. ¿Puede describir su rol en el sistema de detección, notificación y respuesta a brotes?
2. ¿Puede describir sobre cómo fue informado inicialmente del brote?
3. ¿Cuál es su percepción de las acciones tomadas antes de ser notificado del brote?
4. Pensando en el momento en que fue informado del brote, ¿cuál fue su reacción inicial?
5. Después de enterarse de que había un brote, ¿qué acciones tomó?
6. ¿Puede ayudarme a entender por qué tomó estas acciones (por ejemplo, ¿siguió un protocolo? ¿siguió su instinto)?
7. ¿Puede describir sobre los desafíos que enfrentó al tomar estas acciones?
8. ¿Cuál es su percepción sobre las acciones tomadas después de ser notificado del brote?
9. ¿Puede describirme otros problemas que considere importantes abordar para asegurar una respuesta eficiente a un brote?

**English Version**

1. Can you describe your role in the outbreak detection, notification, and response system?
2. Can you walk me through how you were initially informed of the outbreak?
3. What is your perception of the actions taken prior to your notification of the outbreak?
4. Thinking back to the moment you were informed of the outbreak, what was your initial reaction?
5. After you found out there was an outbreak, what actions did you take?
6. Can you help me understand why you took these actions (e.g. did you follow a protocol? did you follow your instinct)?
7. Can you walk me through the challenges you faced in taking these actions?
8. What is your perception on the actions taken following your notification of the outbreak?
9. Can you describe to me any other issues you feel are important to address to ensure an efficient response to an outbreak?
