# Supplementary material for: Bottleneck and enabler evaluation of avian influenza health event — Guatemala, January-February 2023
Source: PLOS Glob Public Health. 2025 Nov 6;5(11):e0005443. doi: 10.1371/journal.pgph.0005443 (PMC12591453; doi:10.1371/journal.pgph.0005443)
Supplement: S1 Table — (DOCX) [file pgph.0005443.s002.docx]

| Date of interview |  |
| --- | --- |
| ID of respondent |  |
| Interviewers |  |
| Organization of respondent |  |
| 5 Quotes |  |
| 5 Surprising moments |  |
| 5 Suggestions from interviewer |  |
| 5 Interventions we identified |  |
| Further questions? |  |

**S1 Table: Rapid Qualitative Methodology Tool**

Analysis Table (1/interview)

Synthesis Table (1/group)

| Organization/Level |  |
| --- | --- |
| Activity |  |
| 3 Most powerful quotes |  |
| 3 things working well |  |
| 3 suggestions |  |
| 3 things need to address |  |
| Detection |  |
| Notification |  |
| Response |  |
