# Supplementary material for: Bottleneck and enabler evaluation of avian influenza health event — Guatemala, January-February 2023
Source: PLOS Glob Public Health. 2025 Nov 6;5(11):e0005443. doi: 10.1371/journal.pgph.0005443 (PMC12591453; doi:10.1371/journal.pgph.0005443)
Supplement: Attachment — Submitted filename: PGPH-D-25-01179_reviewer.pdf [file pgph.0005443.s004.pdf]

PLOS Global Public Health

Bottleneck and Enabler Evaluation of Avian Influenza Health Event — Guatemala, January-February 2023

--Manuscript Draft--

**Manuscript Number:**	PGPH-D-25-01179
**Article Type:**	Research Article
**Full Title:**	Bottleneck and Enabler Evaluation of Avian Influenza Health Event — Guatemala, January-February 2023
**Short Title:**	Evaluation of avian influenza response in Guatemala
**Corresponding Author:**	Parminder S. Suchdev<br>Centers for Disease Control and Prevention<br>Atlanta, Georgia UNITED STATES OF AMERICA
**Order of Authors:**	Parsa Bastani, PhD
	Edgar Bailey Leonardo
	Jose Carlos Monzon Fuentes
	Cesar Conde Pereira
	Emily Zielinski Gutierrez
	Parminder S. Suchdev
**Keywords:**	
**Abstract:**	In February 2023, H5N1 was identified in 11 wild pelicans in Izabal, Guatemala. These were the first known cases of H5N1 in the country. This study assessed the timeliness of the response to this One Health event using the “7-1-7” benchmarks, which propose the following metrics: detection within seven days, notification within one day, and completion of early response within seven days. Open-ended interviews were conducted in September 2023 with nine key informants from the Ministry of Agriculture, Livestock, and Food (MAGA) and the Ministry of Health and Social Assistance (MSPAS) who were directly involved in the response. Participants included epidemiologists, laboratory analysts, and other relevant personnel. Interviews were analyzed using UNICEF’s “Human-Centered Design 4 Health” approach to qualitative fieldwork. Detection and notification were completed in one day, while early response was completed in 34 days. Key enablers of the response included interregional notification and cooperation, availability of earmarked emergency funds, event-based surveillance, and support from laboratories across public and private sectors. Reported bottlenecks included limited national testing capacity, challenges in inter-agency and intra-agency communication, workforce constraints, and equipment shortages. This outbreak response met the detection and notification criteria but did not achieve the 7-day target for completing early response activities. As one of the few qualitative studies examining avian influenza response in Central America, these findings highlight how strengthening a One Health approach, particularly in communication, workforce, and laboratory capacity, could enhance preparedness for future outbreaks.
**Additional Information:**	
**Question**	**Response**
**Financial Disclosure**<br><br>Enter a financial disclosure statement that describes the sources of funding for the work included in this submission and the role the funder(s) played. This includes	This work was funded by the Centers for Disease Control & Prevention

Powered by Editorial Manager® and ProduXion Manager® from Aries Systems Corporation

grants and any commercial funding of the work or authors.<br><br>This statement will be typeset if the manuscript is accepted for publication.<br><br>*Please review the submission guidelines and the instructions link below for detailed requirements and guidance.*	
**Competing Interests**<br><br>On behalf of all authors, disclose any competing interests that could be perceived to bias this work.<br><br>This statement will be typeset if the manuscript is accepted for publication.<br><br>*Please review the instructions link below and PLOS Global Public Health's competing interests policy to determine what information must be disclosed at submission.*	The authors have declared that no competing interests exist.
**Data Availability**<br><br>Before publication, Authors are required to make fully available and without restriction all data underlying their findings. Please see our PLOS Data Policy page for detailed information on this policy.<br><br>A **Data Availability Statement**, detailing where the data can be accessed, is required at first submission. Insert your Data Availability Statement in the box below.<br><br>Please see the data reporting section of our submission guidelines for instructions on what you need to include in your Data Availability Statement.	All relevant data are within the paper.

Powered by Editorial Manager® and ProduXion Manager® from Aries Systems Corporation

This statement will be typeset if the manuscript is accepted for publication.<br><br>*PLOS allows rare exemptions to address legal and ethical concerns. If you have legal or ethical restrictions, please use the box below to detail these in full sentences for the Journal team to consider.*	

Manuscript

Click here to access/download;Manuscript;2025_5_12_Bastani_PLOS

Bottleneck and Enabler Evaluation of Avian Influenza Health Event — Guatemala, January-February 2023

Parsa Bastani[1], Edgar Bailey Leonardo[2], Jose Carlos Monzon Fuentes[2], Cesár Conde Pereira[3], Emily Zielinski Gutierrez[2], Parminder S. Suchdev[2*]

1. Epidemic Intelligence Service, Centers for Disease Control and Prevention, Atlanta, GA 30329, USA
2. Central America Office, Division of Global Health Protection, Centers for Disease Control and Prevention, Guatemala City, Guatemala.
3. Ministry of Public Health and Social Assistance, Guatemala

***Corresponding author:**
Parminder S Suchdev, MD, MPH
U.S. Embassy Guatemala
Boulevard Austracio 11-51, Zone 16, Guatemala City, 01016
psuchdev@cdc.gov

Sources of Support: This work was funded by the Centers for Disease Control & Prevention

Additional disclosure: The findings and conclusions in this report are those of the authors and do not necessarily represent the official position of the US Centers for Disease Control and Prevention.

Data Sharing: Data described in the manuscript, code book, and analytic code will be made available upon request.

Author Contributions: PB, EBL, and PSS designed the study. PB designed the semi-structured interview guide and conduct analyses. PB, EBL and JMF conducted interviews. PB and PSS led the manuscript development and are primarily responsible for the final content. All authors have read and approved the final manuscript.

Running title: Evaluation of avian influenza response in Guatemala

Abstract

In February 2023, H5N1 was identified in 11 wild pelicans in Izabal, Guatemala. These were the first known cases of H5N1 in the country. This study assessed the timeliness of the response to this One Health event using the "7-1-7" benchmarks, which propose the following metrics: detection within seven days, notification within one day, and completion of early response within seven days. Open-ended interviews were conducted in September 2023 with nine key informants from the Ministry of Agriculture, Livestock, and Food (MAGA) and the Ministry of Health and Social Assistance (MSPAS) who were directly involved in the response. Participants included epidemiologists, laboratory analysts, and other relevant personnel. Interviews were analyzed using UNICEF's "Human-Centered Design 4 Health" approach to qualitative fieldwork. Detection and notification were completed in one day, while early response was completed in 34 days. Key enablers of the response included interregional notification and cooperation, availability of earmarked emergency funds, event-based surveillance, and support from laboratories across public and private sectors. Reported bottlenecks included limited national testing capacity, challenges in inter-agency and intra-agency communication, workforce constraints, and equipment shortages. This outbreak response met the detection and notification criteria but did not achieve the 7-day target for completing early response activities. As one of the few qualitative studies examining avian influenza response in Central America, these findings highlight how strengthening a One Health approach, particularly in communication, workforce, and laboratory capacity, could enhance preparedness for future outbreaks.

Introduction

Avian influenza outbreaks are a persistent global health concern, affecting both animal and human populations. Specifically, highly pathogenic avian influenza (HPAI) is a highly contagious disease that can be fatal in birds (1). Humans with close or prolonged, unprotected exposures are at a greater risk of infection. Human illnesses range from mild to severe, sometimes leading to death (2). In 2022 and 2023, outbreaks of H5N1, a strain of HPAI, were identified in both domestic and wild birds as the virus spread among migratory waterfowl from Canada and the United States to Mexico, Central, and South America(3). The virus spread mainly along the Pacific flyway and resulted in the first occurrence of HPAI in Argentina, Bolivia, Colombia, Costa Rica, Cuba, Ecuador, Guatemala, Honduras, Panama, Peru, Uruguay, and Venezuela(4). The increased number of outbreaks in bird populations across this region raised concerns that the virus is becoming more fit and adept at spreading (5).

In January 2023, an outbreak of H5N1was suspected in 11 brown pelicans (*Pelecanus occidentalis*) in Izabal, Guatemala, a coastal department that abuts the Caribbean Sea. Local fisherman noticed these sick pelicans and alerted a regional epidemiologist stationed in Izabal. In collaboration with partners, H5N1 was confirmed, and the Guatemalan government coordinated several actions to contain and manage the outbreak. While there was no human spillover, these cases of H5N1 in Guatemala underscore the need to strengthen health security measures and ensure adequate public health systems are in place to address potential outbreaks.

Specifically, early disease detection and response are necessary to contain HPAI outbreaks and minimize their potential spread to humans. One tool available in helping countries identify gaps in their preparedness systems and strategies are the 7-1-7 metrics(6, 7). These are a set of indicators that measure the timeliness of detection, notification, and early response

activities. The goal of 7-1-7 is to ensure governments can meet the following metrics during an outbreak: detect within 7 days of emergence, report to a public health authority responsible for action within 1 day of detection, and complete early response actions within 7 days from reporting to public health authorities. The metric's authors chose these benchmarks based on a detailed examination of past outbreak timeliness data and the World Health Organization's Integrated Disease Surveillance and Response (IDSR) Technical Guidelines (3rd Edition)(7, 8). This approach supplements the shortcomings of existing pandemic preparedness measures, which often fail to capture how emergency response systems function in real-time. For example, scores on the WHO Joint External Evaluation (JEE) and Global Health Security Index were not correlated with COVID-19 outcomes, such as standardized infection rates (9). To address this gap, in August 2023, the WHO incorporated 7-1-7 targets into its early action review (EAR) guidance to help countries evaluate the timeliness of their early detection and response activities(10).

A qualitative understanding of issues that serve as bottlenecks and enablers for timely outbreak response is key to achieving 7-1-7 targets. We define these targets as the following: detection was when local residents detected the aberrant health event of sick pelicans along the coast; notification was when the event was reported to the Guatemalan Ministry of Agriculture, Livestock and Food; and early response completion was when the investigation team was deployed, initial risk assessments were completed, laboratory confirmation of the outbreak etiology was obtained, and public health measures, risk communication, and community engagement measures were initiated by human and animal health sectors. This information can help governments direct where to channel funding and resources for improving their outbreak preparedness. Thus far, one retrospective study has been published on identifying bottlenecks

and enablers to 7-1-7 system performance (6). In this study, data collection appeared to primarily take place through reviewing situations, intra-action and after-action reviews, discussions with subnational and national-level responders, and a 1-day workshop. Given the limited research on 7-1-7 bottlenecks and enablers, additional evaluation tools are needed.

This study expands upon extant 7-1-7 literature by focusing on a country in Central America, examining an animal outbreak of a zoonotic disease prior to a potential spill-over event, and proposing a unique qualitative methodology for collecting and analyzing data on health emergency preparedness. Specifically, this study systematizes the collection of qualitative materials through semi-structured interviews with key officials. Our aims were twofold: 1) to calculate 7-1-7 timeliness metrics during the January H5N1 outbreak in Guatemala, and 2) to identify bottlenecks and enablers that affected this outbreak response.

Methods

Study design and data collection

Recruitment and interviews were conducted between 17/09/2023 and 30/09/2023 with nine key informants in Guatemala involved in the H5N1 response using a purposive sampling strategy. Key informants were selected from government agencies that were most involved in the outbreak. These included epidemiologists, laboratory analysts, and program officers from the Guatemalan Ministry of Agriculture, Livestock and Food (MAGA) and the Ministry of Public Health and Social Assistance (MSPAS). Due to high staff turnover, it was not possible to locate and consent further respondents within the recruitment time frame. Interviews were conducted in Spanish and English and lasted between 30-60 minutes. Interviews were either conducted on the phone or in-person in a private location in an office space. All respondents were verbally

consented prior to each interview and consent was documented in the written field notes. Each interview was recorded and transcribed.

Data analysis

Analysis of interview data was conducted using the rapid inquiry approach published in United Nations International Children's Emergency Fund's (UNICEF) "Human-Centered Design 4 Health."(11) This toolkit was originally designed to reduce the technical barriers for conducting qualitative research on basic health services and has been used in several published studies(12-15). In other words, UNICEF designed this toolkit so that people with no technical training could perform qualitative research in their communities. We drew on the toolkit's analyses framework using its "Record Field Research" and "Synthesis Tool" worksheets to identify important themes from the transcripts. We followed UNICEF's overall analysis process: first, we used one "Record Field Research" worksheet per interview to analyze each of the interviews. Second, we grouped together the 'Record Field Research' worksheets based on the relevant backgrounds of respondents. In this case, we grouped interviews together based on their respective agency and the level in which they worked (i.e. national or local). Last, we reviewed the completed 'Record Field Research' worksheets for each subgroup in order to fill out one 'Synthesis Tool' worksheet per that subgroup. However, we also adapted the UNICEF HCD Toolkit to better capture data on disease outbreaks. We specifically changed the "Synthesis Tool" worksheet by: replacing references to "immunization services" to "overall outbreak response capacities", collecting three of the most powerful quotes (rather than "3 AHA! Moments") and removing the section on identifying "2 Things We Must Address" to instead highlight the overall group's perspective on detection, notification, and response.

Ethics

This activity was reviewed by the US Centers for Disease Control and Prevention (CDC) and was conducted consistent with applicable federal law and CDC policy. (§See e.g., 45 C.F.R. part 46, 21 C.F.R. part 56; 42 U.S.C. §241(d); 5 U.S.C. §552a; 44 U.S.C. §3501 et seq)

Results

Assessing timeliness

Detection and notification met 7-1-7 timeliness benchmarks; however, the early response did not meet these benchmarks. Detection and notification both took one day. Our records (**Table 1**) indicate that fisherman from Izabal alerted a regional-level epidemiologist from MAGA of 11 sick pelicans on January 25th, 2023, the same day they noticed the pelicans. The epidemiologist arrived at the scene of the health event on January 26th, 2023, and notified that same day the national office of MAGA, the entity responsible for action. In contrast to the timeliness of detection and notification, the early response took approximately 34 days. On January 27th, MAGA sent officials to bring two deceased pelicans from Izabal to Guatemala City for HPAI testing. Immediately after transferring samples, active surveillance and biosafety and biosecurity measures were put in place including animal quarantine measures and decontamination. Once in the custody of MAGA in Guatemala City, samples were sent to National Veterinary Services Laboratories (NVSL) in Ames, Iowa for testing. These samples were received by NVSL on February 6th. The H5N1 confirmation from the NVSL in Ames, Iowa was received on February 6th. The delay in the early response was a result of the inability to coordinate a meeting between the MSPAS and Izabal stakeholders about protocols for dealing with human transmission. While the exact date could not be confirmed, interviews corroborate that this meeting took place, at the earliest, on March 1st.

Table 1: Timeline of significant events in the avian influenza outbreak.

Timeline
January 25th: Regional MAGA epidemiologist receives alert from community of 11 sick pelicans
January 26th: Regional MAGA epidemiologist investigates outbreak and alerts national MAGA
January 27th: MAGA sends officials to bring 2 whole pelicans from Izabal to Guatemala City for testing.
January 28th: Private lab in Guatemala confirms H5 in pelican sample
January 30: Samples of Pelicans sent to National Veterinary Services Laboratories (NVSL) in Ames, Iowa
February 6th: Results received from NVSL of samples positive for H5N1
March 1st: Approximate date for meeting between Ministry of Public Health and Social Assistance (MSPAS) and Izabal stakeholders.

Enablers

Interregional notification and cooperation

Notifications of avian influenza in neighboring countries from the World Animal Health Information System (WAHIS) placed Guatemalan national authorities on high alert for potential HPAI cases in animals. Launched by the World Organization for Animal Health (WOAH), WAHIS is an early warning and monitoring system that disseminates information about animal health and sends alerts on animal diseases and events in real-time (16) . As one official from MAGA stated,

> "Since November, we were expecting to have the first case. The outbreak was not a big surprise, it was part of surveillance. How we managed the case, it was okay."

The respondent is referencing the multiple HPAI outbreaks in animals across Latin and South America between October 2022 and January 2023(17). This knowledge helped the Guatemalan government prepare for a potential outbreak.

Emergency funding

For this health event, there was emergency funding available to carry out epidemiological activities. The funds came from the International Regional Organization for Plant and Animal Health (OIRSA), an organization that aims to develop and coordinate programs for the prevention, control, and eradication of diseases and pests in its member countries (Belize, Costa Rica, Dominican Republic, El Salvador, Guatemala, Honduras, Mexico, Nicaragua, and Panama). The emergency funds from OIRSA are $100,000 per year, and if an outbreak needs additional support, the Regional International Council of Ministries of Agriculture (CIRSA) (OIRSA´s highest authority), can allocate an additional $1 million toward a response. For this emergency, MAGA drew on OIRSA to obtain funds for gasoline, vehicles, per diems, meals, and overnight stays for field teams. The availability of OIRSA funds allowed MAGA to respond to health events without needing to submit requests and obtain approvals for other internal funds. These emergency funds, however, have depleted since the conclusion of this health event. Respondents echoed a "for the permanent appropriation of emergency funds within their nationally approved budgets.

Community event-based surveillance (CEBS)

Community event-based surveillance (CEBS) was pivotal to the Guatemalan's governments detection of H5N1 in pelicans. CEBS is a public health strategy in which community members participate in detecting unusual health events or behaviors (18). Typically, this involves trained surveillance informants identifying and reporting events in the community that have public health significance (19). In Guatemala, community surveillance was developed

empirically through past experiences with disease control strategies, such as the eradication of swine fever (2011-2015) and the management of low-pathogenic avian influenza since the 2000s. During these periods, MAGA personnel identified community leaders to establish a passive surveillance network. As a result, communities know whom to contact and where to report disease suspicions or outbreaks, which facilitated early detection in cases. In Izabal, CEBS was enabled by the strong relationship between community members and the regional level MAGA epidemiological team. As one respondent stated,

> "There is a very good communication between these people [fisherman] and the district epidemiologist. Do you know why? We give vaccines and some medicines. They are very grateful, so any action or thing that happens, they call us. We have a few areas where this communication is hard, the areas where we have more indigenous people. [The indigenous people think that] once you call MAGA, they come to kill [their] animals."

The relationship between the community and government was enabled in part by the demographic and linguistic composition of Izabal. In contrast to other parts of the country, with higher percentages of indigenous populations, government institutions are more easily able to build trust and communicate with the local population in Izabal. According to a 2010 report, Izabal is 25.56 % indigenous(20). In the Puerto Barrios municipality of Izabal, where the pelicans were found, only 3% of the population is indigenous(20). During this outbreak, fisherman from Izabal directly called the regional epidemiologist to inform that person about the 11 dying pelicans. The point of contact for the local fisherman was a MAGA epidemiologist who had established a strong network of contacts after working in the area for 12 years.

Wide network of laboratories

The government's ability to collaborate with private laboratories enabled a timely response. The pelican samples were brought from Izabal to Guatemala City on the evening of Friday January 27th. At this time, the national MAGA laboratories were closed for the weekend and the epidemiological team instead took the samples to Laboratorio Sistemas y Equipos, a private laboratory. At the time, there were 4 veterinary diagnostic laboratories that the Guatemalan government had certified and provided official licenses. A polymerase chain reaction (real-time PCR) test revealed that the samples were positive for Avian Influenza H5 on January 28th, 2023, which was the second day of initiating an early response.

Bottlenecks

National Testing Capacity

Governmental and private laboratories had the reagents to test for H5 but not the N1 subtype in Influenza virus type A (21). Without the adequate diagnostic equipment to confirm the subtype H5N1 within Guatemala, the state's public health system was reliant on reference laboratories in the United States. The samples (on one Flinders Technology Associates® card with the virus isolated) were shipped on Monday January 30th to NVSL in Ames, Iowa, five days after the initial notification on Wednesday, January 25th. MAGA received notification from NSVL on February 6th 2023 that the samples were positive for H5N1. The lag between shipment and notification was due to severe weather conditions in the USA that impacted the courier service.

Workforce Need

Workforce needs were noted by all respondents. For example, a respondent from the Department of Surveillance and Control of the Directorate of Epidemiology and Risk Management in MSPAS stated,

> "the main problem is that there are no human resources, it is the same person in charge of surveillance, the same person who goes to the response, the same person to capacity build the team, the same person to be in the field, it’s a limitation to our resources in general"

As pointed out, the same national-level epidemiologist who oversees surveillance are also in charge of outbreak responses for all respiratory diseases. The lack of adequate, non-emergency funding to bolster staffing slowed down the responsiveness of MSPAS.

PPE Equipment:

This health event highlighted that the workforce lacked equipment and training for addressing pathogenic and contagious diseases in animals. An interviewee from MAGA’s national office was unsure if response teams knew how to don and doff PPE and to safely take and send biological samples. These concerns were echoed at the regional level. One respondent who worked on the ground in Izabal during this health event stated:

> “The truth is that we were not prepared because we did not know what we were facing. The Programa Nacional de Sanidad Avícola (PROSA) response team [that was sent from the national level] had its protective equipment, but it did not have protection for all individuals [involved in the response who lived in the Izabal region]. [Our regional team] didn't have swabs, we didn’t even have coolers [to transport the dead pelicans], something so simple...we weren't prepared, the truth is that the team wasn't prepared for what was coming.”

After voicing these concerns to colleagues at the national level, this respondent was promised additional supplies but was sent 5 pairs of gloves, a bag of straws, and four overalls to address further surveillance and biosecurity response efforts due to resource constraints. Early in the response, the respondent had to reuse gloves while handling the deceased pelicans because they had already run out of clean gloves.

Intra-agency and inter-agency communication and planning

There was a lack of clear communication between animal and human health sectors in Guatemala. The completion of the early response was most impacted by slow coordination between MSPAS and MAGA. MSPAS officials blamed their lack of early response on a delay in notification from MAGA. As one official recounted,

> "One of the issues we complained about is that the new information did not come to us in a timely way…for this reason the response happened a bit late. We would have wanted to know earlier…we wanted the information when they had the suspicion."

From information obtained from interviews, it appears that MSPAS received this notification between 5 and 7 days after the initial notification to MAGA.

However, internal agency communication and planning in MSPAS appeared to be even more key in delaying the response. Once MSPAS received the notification, they did not take any on-the-ground action until at least March 1st. It took MSPAS several weeks to find the personnel, equipment, and funding. Thus, even with the delay in notification from MAGA, there appeared to be unclear lines of communication within MSPAS to initiate a cross-sectoral response that would protect humans against potential disease spillover.

The regional level epidemiological team of MAGA reported that they did not have a clear understanding of their role for both routine surveillance and outbreak response vis-a-vis their counterparts at the national level. Although this did not impact the timeliness of initiating a response, communication issues affected the quality of the response. For this health event, the regional level team worked with the PROSA, a subunit of MAGA that led response efforts. Our respondent at the regional level did not know if PROSA's domain of work included more routine surveillance or if it was limited to responses to health events and outbreaks. When discussing PROSA's response activities, the respondent stated,

> "The next week, another group [from PROSA] came, but no one gave them the information from last week and there was no one who served as their leader. [The week after that], another group [from PROSA] came, but no one gave them the information about the previous week"

This respondent pointed to a disjointed response from PROSA and noted that the area map they were working was outdated. On it were communities that no longer existed or had moved. Not having consulted the regional epidemiological team of MAGA, the early response may have encountered delays, duplications, and a lack of coverage.

Discussion

Timeliness Achievements

Our results indicated that detection and notification met the targets of seven and one days, respectively. However, the completion of the early response far exceeded the 7-day benchmark and instead took approximately 34 days (**Tables 1 and 2**). All steps pertaining to the animal health sector were completed in a timely manner, including deploying

investigation/response teams, conducting epidemiological analysis and an initial risk assessment, obtaining laboratory confirmation of the outbreak etiology, initiating risk communication in affected communities, and establishing a coordination mechanism. The delays in meeting the benchmark were due to   issues coordinating with the human health sector (MSPAS), which took 34 days from the date of notification. If there had been human spillover during this outbreak, containment efforts could have been severely hampered as a result.

Table 2: 7-1-7 Milestone Dates

7-1-7 Milestone Dates	Date	Timeliness (in days)	Target (in Days)	Met Target (Yes/No)
Emergence	25 January 2023	Not Applicable	Not Applicable	Not Applicable
Detection	25 January 2023	0	7	Yes
Notification	26 January 2023	1	1	Yes
Early Response Action Completion	1 March 2023	34	7	No

Recommendations

This study highlighted two obstacles in carrying out 7-1-7 assessments. First, bottleneck and enabler analysis could benefit from a more expansive discussion of methods. The previously published study suggests that investigators review documentation of outbreak response efforts and conduct workshops to gather consensus around bottlenecks and enablers(6). As was the case in our study, there was little publicly available documentation on the outbreak response. For this reason, we relied almost entirely on qualitatively collected data. We chose to focus data collection on interviews due to the ethical challenges of carrying out workshops. Workshops would function similarly to focus groups, a qualitative method in which a group of people are asked to discuss and collectively answer questions posed by a moderator. The problem with avian influenza outbreaks, like many other disease outbreaks, is that they are highly politicized health events (22-24). There are many powerful players placed at risk during an outbreak, such

as tourism industries, livestock companies, and politicians. Given the sensitive climate surrounding outbreaks, we decided that focus groups might hinder an honest dialogue between government stakeholders about bottlenecks. This decision was supported by the literature, which notes that focus groups are not effective if participants fear future reprisal or negative impacts because of their honesty (25, 26). In addition to these suggestions about conducting 7-1-7 assessments, this study's qualitative methodology provides unique insight into specific areas of avian influenza disease surveillance in Guatemala that may serve as roadblocks in future outbreaks. Corroborated by the 7-1-7 bottleneck literature, faltering cooperation between the human and animal health sectors in this avian influenza outbreak was a primary reason for a delay in early response activities(6). In the realm of influenza, this separation of animal and human surveillance is quite common (27, 28). In line with calls from the Pan American Health Organization, evidence from this research suggests that the risks of emerging zoonotic epidemics could be mitigated if all member states (including Guatemala) further enhance coordinated surveillance across the human-animal-environmental interface (29). This One Health approach could more efficiently facilitate coordinated communication, data collection and transfer, and resource allocation.

There are many examples of coordinate One Health surveillance approaches in the literature. In Thailand, a One Health surveillance system was coordinated by a single office (the Coordinating Unit for One Health), which had the task of maintaining interagency communication and presiding over the cross-governmental implementation of One Health activities (30). Taiwan also implements a One Health approach, whereby it integrates wild bird virologic surveillance, domestic avian influenza surveillance, market avian virologic surveillance, swine influenza surveillance, sentinel physician surveillance, human virologic

surveillance, and severe case surveillance (31). These and other models (32) may serve as important starting points for designing a One Health surveillance and outbreak response system.

Efforts to develop a One Health response have been underway in Guatemala. Since 2021, the Centers for Disease Control and Prevention (CDC) have been promoting the One Health approach in Central America, including Guatemala. CDC engaged with the Ministry of Public Health and Social Assistance (MSPAS), the Ministry of Agriculture, Livestock, and Food (MAGA), and the Ministry of Environment and Natural Resources (MARN) to integrate efforts. If the governmental agreement is signed, the One Health Technical Working Group could have an official mandate that facilitates its operation and cooperation among the ministries. This could enable the establishment of more concrete and effective actions for the detection, notification, and response to zoonotic diseases, thereby improving public health in Guatemala. Institutionalization could also strengthen coordination and communication between the human, animal, and environmental health sectors, promoting a faster and more efficient response to health emergencies.

Last, Guatemalan outbreak response capacities could benefit from strengthening diagnostic testing capabilities and workforce. During the health event in January 2023, the national MAGA laboratories did not have the capacity to test for H5N1 and relied on the Pan American Health Organization and the Centers for Disease Control and Prevention to test samples in laboratories outside of Guatemala. Following this incident, gaps were identified in the national animal health laboratory capacities, and improvements were made. Strengthening MSPAS's capacity to test for HPAI would improve 717 metrics and further prepare the agency for outbreaks.

Limitations

There were several limitations to this study. First, the small sample size may introduce biases by skewing the data to the perceptions of those with whom we were able to interview. Second, it was difficult to access MSPAS and MAGA staff the local level, who were involved in this outbreak due to high turnover rates. This information from the local level would have provided an even more fine-tuned understanding of the strengths and drawbacks of the outbreak response. Third, the retrospective nature of this study made it difficult to identify exact dates for calculating 7-1-7 metrics. These recall issues may have made it difficult to identify the exact dates of early response completion.

Despite these limitations, this paper provides insight into the application of 7-1-7 benchmarks to a zoonotic disease and the Guatemalan government's public health emergency response system. By strengthening One Health approaches to public health, guaranteeing funding mechanisms for public health emergencies, and upgrading laboratory capacities, the Guatemalan government may be able to enhance its ability to provide a timely response to future outbreaks. Future use of the 7-1-7 framework could include a larger pool of respondents, a longitudinal data design, or real-time data collection in order to provide greater nuance into outbreak preparedness in Guatemala and elsewhere.

Acknowledgements: The authors would like to thank all the study participants; the Central America Regional Office in the CDC for funding and coordinating this project; the Global Surveillance, Laboratory, and Data Systems Branch, the Global Health Security Team in the CDC's Division for Global Health Protection, and the Influenza Division of the National Center for Immunization and Respiratory Diseases for advising on this project.

References

1. Musa O, Salaudeen A, Akanbi A, Bolarinwa O. Risk Factors, Threats And Prevention Of Highly Pathogenic Avian Influenza (HPAI) In African Countries. African Journal of Clinical and Experimental Microbiology. 2009;10(2).
2. Uyeki TM, Milton S, Abdul Hamid C, Reinoso Webb C, Presley SM, Shetty V, et al. Highly Pathogenic Avian Influenza A(H5N1) Virus Infection in a Dairy Farm Worker. New England Journal of Medicine. 2024;390(21):2028-9.
3. Adlhoch C, Fusaro A, Gonzales JL, Kuiken T, Marangon S, Mirinaviciute G, et al. Avian influenza overview December 2022 – March 2023. EFSA Journal. 2023;21(3).
4. Awada L. Update of the Global and regional epidemiological situation of avian influenza outbreaks. World Organisation for Animal Health; 2023.
5. Harvey J, Mullinax J, Runge M, Prosser D. The changing dynamics of highly pathogenic avian influenza H5N1: Next steps for management & science in North America. Biological Conservation. 2023;282.
6. Bochner AF, Makumbi I, Aderinola O, Abayneh A, Jetoh R, Yemanaberhan RL, et al. Implementation of the 7-1-7 target for detection, notification, and response to public health threats in five countries: a retrospective, observational study. The Lancet Global Health. 2023;11(6):e871-e9.
7. Frieden TR, Lee CT, Bochner AF, Buissonnière M, McClelland A. 7-1-7: an organising principle, target, and accountability metric to make the world safer from pandemics. The Lancet. 2021;398(10300):638-40.
8. Why 7-1-7? The evidence behind the global target for containing epidemic threat. In: Alliance --, editor. p. 1-3.
9. Bollyky TJ, Hulland EN, Barber RM, Collins JK, Kiernan S, Moses M, et al. Pandemic preparedness and COVID-19: an exploratory analysis of infection and fatality rates, and contextual factors associated with preparedness in 177 countries, from Jan 1, 2020, to Sept 30, 2021. The Lancet. 2022;399(10334):1489-512.
10. Guidance and tools for conducting an early action review (EAR): rapid performance improvement for outbreak detection and response. Geneva: World Health Organization; 2023.
11. UNICEF. Human-centered approach for health. 2018.
12. Chaney SC, Mechael P. So Many Choices, How Do I Choose? Considerations for Selecting Digital Health Interventions to Support Immunization Confidence and Demand. Journal of Medical Internet Research. 2023;25:e47713.
13. Hopkins KL, Underwood T, Iddrisu I, Woldemeskel H, Bon HB, Brouwers S, et al. Community-Based Approaches to Increase COVID-19 Vaccine Uptake and Demand: Lessons Learned from Four UNICEF-Supported Interventions. Vaccines. 2023;11(7):1180.
14. Ivanova V, Shahabuddin ASM, Sharkey A, Johri M. Advancing Immunization Coverage and Equity: A Structured Synthesis of Pro-Equity Strategies in 61 Gavi-Supported Countries. Vaccines. 2023;11(1):191.
15. Jalloh MF, Patel P, Sutton R, Kulkarni S, Toure M, Wiley K, et al. Qualitative assessment of caregiver experiences when navigating childhood immunisation in urban communities in Sierra Leone. BMJ Open. 2022;12(5):e058203.
16. World Animal Health Information System [Available from: https://www.woah.org/en/what-we-do/animal-health-and-welfare/disease-data-collection/world-animal-health-information-system/.
17. Organization PAH. Epidemiological Update: Outbreaks of avian influenza caused by influenza A(H5N1) in the Region of the Americas. PAHO; 2023.

18. Clara A, Dao ATP, Do TT, Tran PD, Tran QD, Ngu ND, et al. Factors Influencing Community Event-based Surveillance: Lessons Learned from Pilot Implementation in Vietnam. Health Security. 2018;16(S1):S-66-S-75.
19. Technical Guidelines for Integrated Disease Surveillance and Response in the African Region. Brazzaville: World Health Organization. Regional Office for Africa; 2010.
20. Plan De Desarrollo Departamental 2011-2025. Secretaria de Planificacion y Programacion de la Presidencia de Izabal; 2010.
21. WHO information forthe molecular detectionof influenza viruses. The World Health Organization; 2021.
22. Vu T. Epidemics as Politics with Case Studies from Malaysia, Thailand, and Vietnam. Global Health Governance. 2011;IV(2).
23. Ruan LK, Jeffrey; Crete-Nishihata, Masashi. Censored Contagion: How Information on the Coronovirus is Managed on Chinese Social Media. 2020.
24. Shir-Raz Y, Elisha E, Martin B, Ronel N, Guetzkow J. Censorship and Suppression of Covid-19 Heterodoxy: Tactics and Counter-Tactics. Minerva. 2023;61(3):407-33.
25. Stalmeijer RE, McNaughton N, Van Mook WNKA. Using focus groups in medical education research: AMEE Guide No. 91. Medical Teacher. 2014;36(11):923-39.
26. Kruegar RC, Mary Anne. Designing and Conducting Focus Group Interviews. In: Kruegar rC, Mary Anne; Donner, Jonathan; Kirsch, Stuart; Maack, Jonathan, editor. Social Analysis: Selected Tools and Techniques. Washington D.C.: The World Bank; 2001. p. 4-23.
27. Chien Y-J. How Did International Agencies Perceive the Avian Influenza Problem? The Adoption and Manufacture of the ‘One World, One Health’ Framework. In: Dingwall R, Hoffman L, Staniland K, editors. Pandemics and Emerging Infectious Diseases: The Sociological Agenda :The Sociological Agenda: John Wiley & Sons Ltd; 2013. p. 46-58.
28. Hinjoy S, Wongkumma A, Kongyu S, Smithsuwan P, Suangtho P, Yingyong T, et al. An Assessment of Epidemiology Capacity in a One Health Team at the Provincial Level in Thailand. Veterinary Sciences. 2016;3(4):30.
29. Organization PAH. ONE HEALTH - Action Track 2 [Available from: https://www.paho.org/en/one-health-action-track-2.
30. Innes GK, Lambrou AS, Thumrin P, Thukngamdee Y, Tangwangvivat R, Doungngern P, et al. Enhancing global health security in Thailand: Strengths and challenges of initiating a One Health approach to avian influenza surveillance. One Health. 2022;14.
31. King C-C, Kao C-L, d D-PL, Cheng M-C, Yen H-L, Lee M-S, et al. Seven integrated influenza surveillance systems in Taiwan. International Congress Series. 2001;1219:107-18.
32. Bordier M, Binot TU-AA, Hendrikx P, Goutard FL. Characteristics of One Health surveillance systems: A systematic literature review. Preventive Veterinary Medicine. 2020;181.
