# Supplementary material for: Bottleneck and enabler evaluation of avian influenza health event — Guatemala, January-February 2023
Source: PLOS Glob Public Health. 2025 Nov 6;5(11):e0005443. doi: 10.1371/journal.pgph.0005443 (PMC12591453; doi:10.1371/journal.pgph.0005443)
Supplement: Attachment — Submitted filename: PGPH-D-25-01179_R1.pdf [file pgph.0005443.s006.pdf]

PLOS Global Public Health

Bottleneck and Enabler Evaluation of Avian Influenza Health Event — Guatemala, January-February 2023

--Manuscript Draft--

**Manuscript Number:**	PGPH-D-25-01179R1
**Article Type:**	Research Article
**Full Title:**	Bottleneck and Enabler Evaluation of Avian Influenza Health Event — Guatemala, January-February 2023
**Short Title:**	Evaluation of avian influenza response in Guatemala
**Corresponding Author:**	Parminder S. Suchdev<br>Centers for Disease Control and Prevention<br>Atlanta, Georgia UNITED STATES OF AMERICA
**Order of Authors:**	Parsa Bastani, PhD
	Edgar Bailey Leonardo
	Jose Carlos Monzon Fuentes
	Cesar Conde Pereira
	Emily Zielinski Gutierrez
	Parminder S. Suchdev
**Keywords:**	
**Abstract:**	In February 2023, H5N1 was identified in 11 wild pelicans in Izabal, Guatemala. These were the first known cases of H5N1 in the country. This study assessed the timeliness of the response to this One Health event using the “7-1-7” benchmarks, which propose the following metrics: detection within seven days, notification within one day, and completion of early response within seven days. Open-ended interviews were conducted in September 2023 with nine key informants from the Ministry of Agriculture, Livestock, and Food (MAGA) and the Ministry of Health and Social Assistance (MSPAS) who were directly involved in the response. Participants included epidemiologists, laboratory analysts, and other relevant personnel. Interviews were analyzed using UNICEF’s “Human-Centered Design 4 Health” approach to qualitative fieldwork. Detection and notification were completed in one day, while early response was completed in 34 days. Key enablers of the response included interregional notification and cooperation, availability of earmarked emergency funds, event-based surveillance, and support from laboratories across public and private sectors. Reported bottlenecks included limited national testing capacity, challenges in inter-agency and intra-agency communication, workforce constraints, and equipment shortages. This outbreak response met the detection and notification criteria but did not achieve the 7-day target for completing early response activities. As one of the few qualitative studies examining avian influenza response in Central America, these findings highlight how strengthening a One Health approach, particularly in communication, workforce, and laboratory capacity, could enhance preparedness for future outbreaks.
**Additional Information:**	
**Question**	**Response**
**Financial Disclosure**<br><br>Enter a financial disclosure statement that describes the sources of funding for the work included in this submission and the role the funder(s) played. This includes	This work was funded by the Centers for Disease Control & Prevention

Powered by Editorial Manager® and ProduXion Manager® from Aries Systems Corporation

grants and any commercial funding of the work or authors.<br><br>This statement will be typeset if the manuscript is accepted for publication.<br><br>*Please review the submission guidelines and the instructions link below for detailed requirements and guidance.*	
**Competing Interests**<br><br>On behalf of all authors, disclose any competing interests that could be perceived to bias this work.<br><br>This statement will be typeset if the manuscript is accepted for publication.<br><br>*Please review the instructions link below and PLOS Global Public Health's competing interests policy to determine what information must be disclosed at submission.*	The authors have declared that no competing interests exist.
**Data Availability**<br><br>Before publication, Authors are required to make fully available and without restriction all data underlying their findings. Please see our PLOS Data Policy page for detailed information on this policy.<br><br>A **Data Availability Statement**, detailing where the data can be accessed, is required at first submission. Insert your Data Availability Statement in the box below.<br><br>Please see the data reporting section of our submission guidelines for instructions on what you need to include in your Data Availability Statement.	All relevant data are within the paper.

Powered by Editorial Manager® and ProduXion Manager® from Aries Systems Corporation

This statement will be typeset if the manuscript is accepted for publication.<br><br>*PLOS allows rare exemptions to address legal and ethical concerns. If you have legal or ethical restrictions, please use the box below to detail these in full sentences for the Journal team to consider.*	

Powered by Editorial Manager® and ProduXion Manager® from Aries Systems Corporation

Manuscript
Click here to access/download;Manuscript;2025_7_28_clean.docx

Bottleneck and Enabler Evaluation of Avian Influenza Health Event — Guatemala, January-February 2023

Parsa Bastani[1], Edgar Bailey Leonardo[2], Jose Carlos Monzon Fuentes[2], Cesár Conde Pereira[3], Emily Zielinski Gutierrez[2], Parminder S. Suchdev[2*]

1. Epidemic Intelligence Service, Centers for Disease Control and Prevention, Atlanta, GA 30329, USA
2. Central America Office, Division of Global Health Protection, Centers for Disease Control and Prevention, Guatemala City, Guatemala.
3. Ministry of Public Health and Social Assistance, Guatemala

***Corresponding author:**
Parminder S Suchdev, MD, MPH
U.S. Embassy Guatemala
Boulevard Austracio 11-51, Zone 16, Guatemala City, 01016
psuchdev@cdc.gov

Sources of Support: This work was funded by the Centers for Disease Control & Prevention

Additional disclosure: The findings and conclusions in this report are those of the authors and do not necessarily represent the official position of the US Centers for Disease Control and Prevention.

Data Sharing: Data described in the manuscript, code book, and analytic code will be made available upon request.

Author Contributions: PB, EBL, and PSS designed the study. PB designed the semi-structured interview guide and conducted analyses. PB, EBL and JMF conducted interviews. PB and PSS led the manuscript development and are primarily responsible for the final content. All authors have read and approved the final manuscript.

Running title: Evaluation of avian influenza response in Guatemala

Abstract

In February 2023, H5N1 was identified in 11 wild pelicans in Izabal, Guatemala. These were the first known cases of H5N1 in the country. This study assessed the timeliness of the response to this One Health event using the “7-1-7” benchmarks, which propose the following metrics: detection within seven days, notification within one day, and completion of early response within seven days. Open-ended interviews were conducted in September 2023 with nine key informants from the Ministry of Agriculture, Livestock, and Food (MAGA) and the Ministry of Health and Social Assistance (MSPAS) who were directly involved in the response. Participants included epidemiologists, laboratory analysts, and other relevant personnel. Interviews were analyzed using UNICEF’s “Human-Centered Design 4 Health” approach to qualitative fieldwork. Detection and notification were completed in one day, while early response was completed in 34 days. Key enablers of the response included interregional notification and cooperation, availability of earmarked emergency funds, event-based surveillance, and support from laboratories across public and private sectors. Reported bottlenecks included limited national laboratory testing capacity, challenges in inter-agency and intra-agency communication, workforce constraints, and equipment shortages. This outbreak response met the detection and notification criteria but did not achieve the 7-day target for completing early response activities. As one of the few qualitative studies examining avian influenza response in Central America, these findings highlight how strengthening a One Health approach, particularly in communication, workforce, and laboratory capacity, could enhance preparedness for future outbreaks.

Introduction

Avian influenza outbreaks are a persistent global health concern, affecting both animal and human populations. Specifically, highly pathogenic avian influenza (HPAI) is a highly contagious disease that can be fatal in birds (1). Humans with close or prolonged, unprotected exposures are at a greater risk of infection. Human illnesses range from mild to severe, sometimes leading to death (2). In 2022 and 2023, outbreaks of H5N1, a strain of HPAI, were identified in both domestic and wild birds as the virus spread among migratory waterfowl from Canada and the United States to Mexico, Central, and South America(3). The virus spread mainly along the Pacific flyway and resulted in the first occurrence of HPAI in Argentina, Bolivia, Colombia, Costa Rica, Cuba, Ecuador, Guatemala, Honduras, Panama, Peru, Uruguay, and Venezuela(4). The increased number of outbreaks in bird populations across this region raised concerns that the virus is becoming more fit and adept at spreading (5).

In January 2023, an outbreak of H5N1was suspected in 11 brown pelicans (*Pelecanus occidentalis*) in Izabal, Guatemala, a coastal department that abuts the Caribbean Sea. Local fisherman noticed these sick pelicans and alerted a regional epidemiologist stationed in Izabal. In collaboration with partners, H5N1 was confirmed, and the Guatemalan government coordinated several actions to contain and manage the outbreak. While there was no human spillover, these cases of H5N1 in Guatemala underscore the need to strengthen health security measures and ensure adequate public health systems are in place to address potential outbreaks.

Specifically, early disease detection and response are necessary to contain HPAI outbreaks and minimize their potential spread to humans. One tool available in helping countries identify gaps in their preparedness systems and strategies is the 7-1-7 metrics(6, 7). This is a set of indicators that measure the timeliness of detection, notification, and early response activities.

The goal of 7-1-7 is to ensure governments can meet the following metrics during an outbreak: detect within 7 days of emergence, report to a public health authority responsible for action within 1 day of detection, and complete early response actions within 7 days from reporting to public health authorities. The metric's authors chose these benchmarks based on a detailed examination of past outbreak timeliness data and the World Health Organization's Integrated Disease Surveillance and Response (IDSR) Technical Guidelines (3rd Edition)(7, 8). This approach helps rectify the shortcomings of existing pandemic preparedness measures, which often fail to capture how emergency response systems function in real-time. For example, scores on the WHO Joint External Evaluation (JEE) and Global Health Security Index were not correlated with COVID-19 outcomes, such as standardized infection rates (9). To address this gap, in August 2023, the WHO incorporated 7-1-7 targets into its early action review (EAR) guidance to help countries evaluate the timeliness of their early detection and response activities(10).

A qualitative understanding of issues that serve as bottlenecks and enablers for timely outbreak response is key to achieving 7-1-7 targets. We define these targets as the following: detection was when local residents detected the aberrant health event of sick pelicans along the coast; notification was when the event was reported to the Guatemalan Ministry of Agriculture, Livestock and Food; and early response completion was when the investigation team was deployed, initial risk assessments were completed, laboratory confirmation of the outbreak etiology was obtained, and public health measures, risk communication, and community engagement measures were initiated by human and animal health sectors. This information can help governments direct where to channel funding and resources for improving their outbreak preparedness. Thus far, one retrospective study has been published on identifying bottlenecks

and enablers to 7-1-7 system performance (6). In this study, data collection primarily took place through reviewing situations, intra-action and after-action reviews, discussions with subnational and national-level responders, and a 1-day workshop. Given the limited research on 7-1-7 bottlenecks and enablers, additional tools are needed for evaluating outbreak situations with limited written records that use the 7-1-7 framework.

This study expands upon extant 7-1-7 literature by focusing on a country in Central America, examining an animal outbreak of a zoonotic disease prior to a potential spill-over event, and proposing a unique, simple, and rapid qualitative methodology for collecting and analyzing data on health emergency preparedness. Specifically, this study systematizes the collection of qualitative materials through semi-structured interviews with key officials. Our aims were twofold: 1) to calculate 7-1-7 timeliness metrics during the January H5N1 outbreak in Guatemala, and 2) to identify bottlenecks and enablers that affected this outbreak response.

Methods

Study design and data collection

Recruitment and interviews were conducted between 17/09/2023 and 30/09/2023 with nine key informants in Guatemala involved in the H5N1 response using a purposive sampling strategy. Key informants were selected from government agencies that were most involved in the outbreak. These included epidemiologists, laboratory analysts, and program officers from the Guatemalan Ministry of Agriculture, Livestock and Food (MAGA) and the Ministry of Public Health and Social Assistance (MSPAS). Due to high staff turnover, it was not possible to locate and consent further respondents within the recruitment time frame. Interviews were conducted in Spanish and English and lasted between 30-60 minutes. Interviews were conducted either on the

phone or in-person in a private location in an office space by PB (an epidemiologist and medical anthropologist) and ELB (an epidemiologist and veterinarian). All respondents were verbally consented prior to each interview and consent was documented in the written field notes. Semi-structured interview guides were administered to each respondent (see Supplement I). Questions covered the research participant's experience and perceptions of the outbreak response during the detection, notification, and response stages. Each interview was recorded and transcribed.

Data analysis

Analysis of interview data was conducted using the rapid inquiry approach published in United Nations International Children's Emergency Fund's (UNICEF) "Human-Centered Design 4 Health."(11) This toolkit was originally designed to reduce the technical barriers for conducting qualitative research on basic health services and has been used in several published studies(12-15). In other words, UNICEF designed this toolkit so that people with no technical training could perform qualitative research in their communities.

We drew on the toolkit's analyses framework using its "Record Field Research" and "Synthesis Tools" worksheets to identify important themes from the transcripts. We followed UNICEF's overall analysis process: first, we filled out one "Record Field Research" worksheet per interview to analyze each of the interviews and second, we filled out one "Synthesis Tool" per interview subgroup. The first step comprised of listing the following for each interview: five meaningful quotes, five surprising moments, five suggestions from the interviewer, and five interventions the analyzer (PB and EBL) identified based on the interview (see supplement II). Second, we placed 'Record Field Research' worksheets into subgroups based on the relevant backgrounds of respondents. Accordingly, we created four subgroups: 1) national level MAGA

staff; 2) local level MAGA staff; 3) national level MSPAS staff, and 4) local level MSPAS staff. Last, we completed one "Synthesis Tool" worksheet for each subgroup by reviewing the 'Record Field Research' worksheets for that subgroup. This comprised of listing the following per subgroup: three powerful quotes, three things that worked well, three suggestions, three specific issues that need to be addressed, and the subgroup's perception of the overall detection, notification, and response to the outbreak (see supplement II). In the UNICEF methodology, the completion of this "Synthesis Tool" worksheet constitutes the analysis process, which equates to the analysis of codes in more conventional qualitative methodologies.

The information captured in the tools we used were adapted from the UNICEF Human Centered Design (HCD) Toolkit to better capture data on disease outbreaks. We specifically changed the "Synthesis Tool" worksheet by: replacing references to "immunization services" to "overall outbreak response capacities", collecting three of the most powerful quotes (rather than "3 AHA! Moments") and removing the section on identifying "2 Things We Must Address" to instead highlight the overall group's perspective on detection, notification, and response. Even though the primary author is a trained qualitative researcher, the research team strategically chose to use the UNICEF toolkit adaptation in order to build local capacity for qualitative analysis. The goal was to not only train local CDC staff in qualitative methods, but also to provide a clear, tested, and simple qualitative analysis tool to other CDC field offices seeking to carryout 7-1-7 analyses.

Ethics

This activity was reviewed by the US Centers for Disease Control and Prevention (CDC) and was conducted consistent with applicable federal law and CDC policy. (§See e.g., 45 C.F.R. part 46, 21 C.F.R. part 56; 42 U.S.C. §241(d); 5 U.S.C. §552a; 44 U.S.C. §3501 et seq)

Results

Assessing timeliness

Detection and notification met 7-1-7 timeliness benchmarks; however, the early response did not meet these benchmarks. Detection and notification both took one day. Our records (**Table 1**) indicate that fisherman from Izabal alerted a regional-level epidemiologist from MAGA of 11 sick pelicans on January 25th, 2023, the same day they noticed the pelicans. The epidemiologist arrived at the scene of the health event on January 26th, 2023, and notified that same day the national office of MAGA, the entity responsible for action. In contrast to the timeliness of detection and notification, the early response took approximately 34 days. On January 27th, MAGA sent officials to bring two deceased pelicans from Izabal to Guatemala City for HPAI testing. Immediately after transferring samples, active surveillance and biosafety and biosecurity measures were put in place including animal quarantine measures and decontamination. Once in the custody of MAGA in Guatemala City, samples were sent to National Veterinary Services Laboratories (NVSL) in Ames, Iowa for testing. These samples were received by NVSL on February 6th. The H5N1 confirmation from the NVSL in Ames, Iowa was received on February 6th. The delay in the early response was a result of the inability to coordinate a meeting between the MSPAS and Izabal community members about protocols for dealing with human transmission. This is a necessary step in the early response because community members should be made aware of how to identify and respond to H5N1 human

spillover as soon as possible. While the exact date could not be confirmed, interviews corroborated that this meeting took place, at the earliest, on March 1st.

Table 1: Timeline of significant events in the avian influenza outbreak

**Timeline**			
**Response Stage**	**Date**	**Response Stage**	**Activity**
**Detection**	January 25th	**Detection**	Regional MAGA epidemiologist receives alert from community of 11 sick pelicans
**Notification**	January 26th	**Notification**	Regional MAGA epidemiologist investigates outbreak and alerts national MAGA
**Early Response**	January 27th	**Early Response**	MAGA sends officials to bring 2 whole pelicans from Izabal to Guatemala City for testing.
	January 28th		Private lab in Guatemala confirms H5 in pelican sample
	January 30th		Samples of Pelicans sent to National Veterinary Services Laboratories (NVSL) in Ames, Iowa
	February 6th		Results received from NVSL of samples positive for H5N1
	March 1st		Approximate date for meeting between Ministry of Public Health and Social Assistance (MSPAS) and Izabal stakeholders.

Enablers

Interregional notification and cooperation

Notifications of avian influenza in neighboring countries from the World Animal Health Information System (WAHIS) placed Guatemalan national authorities on high alert for potential HPAI cases in animals. Launched by the World Organization for Animal Health (WOAH), WAHIS is an early warning and monitoring system that disseminates information about animal health and sends alerts on animal diseases and events in real-time (16) . As one official from MAGA stated,

> "Since November, we were expecting to have the first case. The outbreak was not a big surprise, it was part of surveillance. How we managed the case, it was okay."

The respondent is referencing the multiple HPAI outbreaks in animals across Latin and South America between October 2022 and January 2023(17). This knowledge helped the Guatemalan government prepare for a potential outbreak.

Emergency funding

For this health event, there was emergency funding available to carry out epidemiological activities. The funds came from the International Regional Organization for Plant and Animal Health (OIRSA), an organization that aims to develop and coordinate programs for the prevention, control, and eradication of diseases and pests in its member countries (Belize, Costa Rica, Dominican Republic, El Salvador, Guatemala, Honduras, Mexico, Nicaragua, and Panama). The emergency funds from OIRSA are $100,000 per year, and if an outbreak needs additional support, the Regional International Council of Ministries of Agriculture (CIRSA) (OIRSA´s highest authority), can allocate an additional $1 million toward a response. For this emergency, MAGA drew on OIRSA to obtain funds for gasoline, vehicles, per diems, meals, and overnight stays for field teams. The availability of OIRSA funds allowed MAGA to respond to

health events without needing to submit requests and obtain approvals for other internal funds. These emergency funds, however, have depleted since the conclusion of this health event. Respondents echoed a call for “for the permanent appropriation of emergency funds within their nationally approved budgets.

Community event-based surveillance (CEBS)

Community event-based surveillance (CEBS) was pivotal to the Guatemalan government’s detection of H5N1 in pelicans. CEBS is a public health strategy in which community members participate in detecting unusual health events or behaviors (18). Typically, this involves trained surveillance informants identifying and reporting events in the community that have public health significance (19). In Guatemala, community surveillance was developed empirically through past experiences with disease control strategies, such as the eradication of swine fever (2011-2015) and the management of low-pathogenic avian influenza since the 2000s. During these periods, MAGA personnel identified community leaders to establish a passive surveillance network. As a result, communities know whom to contact and where to report disease suspicions or outbreaks, which facilitated early detection in cases. In Izabal, CEBS was enabled by the strong relationship between community members and the regional level MAGA epidemiological team. As one respondent stated,

> “There is a very good communication between these people [fisherman] and the district epidemiologist. Do you know why? We give vaccines and some medicines. They are very grateful, so any action or thing that happens, they call us. We have a few areas where this communication is hard, the areas where we have more indigenous people.

[The indigenous people think that] once you call MAGA, they come to kill [their] animals."

The relationship between the community and government was enabled in part by the demographic and linguistic composition of Izabal of Spanish speaking, primarily non-Indigenous populations. In contrast to other parts of the country, with higher percentages of indigenous populations, government institutions are more easily able to build trust and communicate with the local population in Izabal. According to a 2010 report, Izabal is 25.56 % indigenous(20). In the Puerto Barrios municipality of Izabal, where the pelicans were found, only 3% of the population is indigenous(20). During this outbreak, fisherman from Izabal directly called the regional epidemiologist to inform that person about the 11 dying pelicans. The point of contact for the local fisherman was a MAGA epidemiologist who had established a strong network of contacts after working in the area for 12 years.

Wide network of laboratories

The government's ability to collaborate with private laboratories enabled a timely response. The pelican samples were brought from Izabal to Guatemala City on the evening of Friday January 27th. At this time, the national MAGA laboratories were closed for the weekend and the epidemiological team instead took the samples to Laboratorio Sistemas y Equipos, a private laboratory. At the time, there were 4 veterinary diagnostic laboratories that the Guatemalan government had certified and provided official licenses. A polymerase chain reaction (real-time PCR) test revealed that the samples were positive for Avian Influenza H5 on January 28th, 2023, which was the second day of initiating an early response.

Bottlenecks

National Testing Capacity

Governmental and private laboratories had the reagents to test for H5 but not the N1 subtype in Influenza virus type A (21). Without the adequate diagnostic equipment to confirm the subtype H5N1 within Guatemala, the state's public health system was reliant on reference laboratories in the United States. The samples (on one Flinders Technology Associates® card with the virus isolated) were shipped on Monday January 30th to NVSL in Ames, Iowa, five days after the initial notification on Wednesday, January 25th. MAGA received notification from NSVL on February 6th 2023 that the samples were positive for H5N1. The lag between shipment and notification was due to severe weather conditions in the USA that impacted the courier service.

Workforce Need

Workforce needs were noted by all respondents. For example, a respondent from the Department of Surveillance and Control of the Directorate of Epidemiology and Risk Management in MSPAS stated,

> "the main problem is that there are no human resources, it is the same person in charge of surveillance, the same person who goes to the response, the same person to capacity build the team, the same person to be in the field, it's a limitation to our resources in general"

As pointed out, the same national-level epidemiologist who oversees surveillance is also in charge of outbreak responses for all respiratory diseases. The lack of adequate, non-emergency funding to bolster staffing slowed down the responsiveness of MSPAS.

PPE Equipment:

This health event highlighted that the workforce lacked equipment and training for addressing pathogenic and contagious diseases in animals. An interviewee from MAGA's national office was unsure if response teams knew how to don and doff PPE and to safely take and send biological samples. These concerns were echoed at the regional level. One respondent who worked on the ground in Izabal during this health event stated:

> "The truth is that we were not prepared because we did not know what we were facing. The Programa Nacional de Sanidad Avícola (PROSA) response team [that was sent from the national level] had its protective equipment, but it did not have protection for all individuals [involved in the response who lived in the Izabal region]. [Our regional team] didn't have swabs, we didn't even have coolers [to transport the dead pelicans], something so simple...we weren't prepared, the truth is that the team wasn't prepared for what was coming."

After voicing these concerns to colleagues at the national level, this respondent was promised additional supplies but was sent 5 pairs of gloves, a bag of straws, and four overalls to address further surveillance and biosecurity response efforts due to resource constraints. Early in the response, the respondent had to reuse gloves while handling the deceased pelicans because they had already run out of clean gloves.

Intra-agency and inter-agency communication and planning

During H5N1 outbreaks, human health sectors should be involved in the response to ensure human spillover can be contained. During this health event, human health sector involvement was the largest barrier to early response completion. One MSPAS official blamed

the lack of timely notification from MAGA as the primary reason for the delay in action. As this official recounted,

> "One of the issues we complained about is that the new information did not come to us in a timely way…for this reason the response happened a bit late. We would have wanted to know earlier…we wanted the information when they had the suspicion."

From information obtained from interviews, it appears that MSPAS received this notification between 5 and 7 days after the initial notification to MAGA.

However, our findings indicate that internal communication and planning within MSPAS played even a bigger role in the delayed response. Although MSPAS received the notification, they did not take any on-the-ground action until at least March 1st, when a meeting was held with community members and officials in Izabal. Due to insufficient protocols and preparedness for One Health emergency situations, it took MSPAS several weeks to mobilize the necessary personnel, equipment, and funding. In short, MSPAS was under-resourced and unprepared to respond effectively to the health event. Thus, even with the initial delay in notification from MAGA, there appeared to be significant gaps in internal coordination and limited capacity within MSPAS to initiate a timely, cross-sectoral response aimed at preventing potential disease spillover to humans.

The regional level epidemiological team of MAGA reported that they did not have a clear understanding of their role for both routine surveillance and outbreak response vis-a-vis their counterparts at the national level. Although this did not impact the timeliness of initiating a response, communication issues affected the quality of the response. For this health event, the regional level team worked with the PROSA, a subunit of MAGA that led response efforts. Our respondent at the regional level did not know if PROSA's domain of work included more routine

surveillance or if it was limited to responses to health events and outbreaks. When discussing PROSA's response activities, the respondent stated,

> "The next week, another group [from PROSA] came, but no one gave them the information from last week and there was no one who served as their leader. [The week after that], another group [from PROSA] came, but no one gave them the information about the previous week"

This respondent pointed to a disjointed response from PROSA and noted that the area map they were working with was outdated. On it were communities that no longer existed or had moved. Not having consulted the regional epidemiological team of MAGA, the early response may have encountered delays, duplications, and a lack of coverage.

Discussion

Timeliness Achievements

Our results indicated that detection and notification met the targets of seven and one days, respectively. However, the completion of the early response far exceeded the 7-day benchmark and instead took approximately 34 days (**Tables 1 and 2**). All steps pertaining to the animal health sector were completed in a timely manner, including deploying investigation/response teams, conducting epidemiological analysis and an initial risk assessment, obtaining laboratory confirmation of the outbreak etiology, initiating risk communication in affected communities, and establishing a coordination mechanism. The delays in meeting the benchmark were due to issues coordinating with the human health sector (MSPAS), which took 34 days from the date of notification. If there had been human spillover during this outbreak, containment efforts could have been severely hampered as a result.

Table 2: 7-1-7 Milestone Dates

7-1-7 Milestone Dates	Date	Timeliness (in days)	Target (in Days)	Met Target (Yes/No)
Emergence	25 January 2023	Not Applicable	Not Applicable	Not Applicable
Detection	25 January 2023	0	7	Yes
Notification	26 January 2023	1	1	Yes
Early Response Action Completion	1 March 2023	34	7	No

Recommendations

This study highlighted two obstacles in carrying out 7-1-7 assessments. First, bottleneck and enabler analysis could benefit from additional qualitative methods. The previously published study suggests that investigators review documentation of outbreak response efforts and conduct workshops to gather consensus around bottlenecks and enablers(6). As was the case in our study, there was little publicly available documentation on the outbreak response. For this reason, we relied almost entirely on qualitatively collected data. We chose to focus data collection on interviews due to the ethical challenges of carrying out workshops. Workshops would function similarly to focus groups, a qualitative method in which a group of people are asked to discuss and collectively answer questions posed by a moderator. The problem with avian influenza outbreaks, like many other disease outbreaks, is that they are highly politicized health events (22-24). There are many powerful players placed at risk during an outbreak, such as tourism industries, livestock companies, and politicians. Given the sensitive climate surrounding outbreaks, we decided that focus groups might hinder an honest dialogue between government stakeholders about bottlenecks. This decision was supported by the literature, which notes that focus groups are not effective if participants fear future reprisal or negative impacts because of their honesty (25, 26).

In addition to these suggestions about conducting 7-1-7 assessments, this study's qualitative methodology provides unique insight into the enablers and roadblocks to avian influenza disease surveillance and preparedness in Guatemala. This analysis confirmed that multinational disease reporting systems (e.g. WAHIS), CEBS, and private-public lab partnerships can enable efficient outbreak detection (27-33). In contrast, , this study also corroborated literature that faltering cooperation between the human and animal health sectors can delay early response activities in outbreak settings(6). In the realm of influenza, this separation of animal and human surveillance is quite common (27, 28). In line with calls from the Pan American Health Organization, evidence from this research suggests that the risks of emerging zoonotic epidemics could be mitigated if all member states (including Guatemala) further enhance coordinated surveillance across the human-animal-environmental interface (29). This One Health approach could more efficiently facilitate coordinated communication, data collection and transfer, and resource allocation.

There are many examples of well-coordinated One Health surveillance approaches in the literature. In Thailand, a One Health surveillance system was coordinated by a single office (the Coordinating Unit for One Health), which had the task of maintaining interagency communication and presiding over the cross-governmental implementation of One Health activities (30). Taiwan also implements a One Health approach, whereby it integrates wild bird virologic surveillance, domestic avian influenza surveillance, market avian virologic surveillance, swine influenza surveillance, sentinel physician surveillance, human virologic surveillance, and severe case surveillance (31). These and other models (32) may serve as important starting points for designing a One Health surveillance and outbreak response system.

Efforts to develop a One Health response have been underway in Guatemala. Since 2021, the Centers for Disease Control and Prevention (CDC) have been promoting the One Health approach in Central America, including Guatemala. CDC engaged with the Ministry of Public Health and Social Assistance (MSPAS), the Ministry of Agriculture, Livestock, and Food (MAGA), and the Ministry of Environment and Natural Resources (MARN) to integrate efforts. If the governmental agreement is signed, the One Health Technical Working Group could have an official mandate that facilitates its operation and cooperation among the ministries. This could enable the establishment of more concrete and effective actions for the detection, notification, and response to zoonotic diseases, thereby improving public health in Guatemala. Institutionalization could also strengthen coordination and communication between the human, animal, and environmental health sectors, promoting a faster and more efficient response to health emergencies.

Last, Guatemalan outbreak response capacities could benefit from strengthening diagnostic testing capabilities and workforce. During the health event in January 2023, the national MAGA laboratories did not have the capacity to test for H5N1 and relied on the Pan American Health Organization and the Centers for Disease Control and Prevention to test samples in laboratories outside of Guatemala. Following this incident, gaps in testing capacity were identified in the national animal health laboratory capacities, and improvements were made. Strengthening MSPAS's capacity to test for HPAI would improve 7-1-7 metrics and further prepare the agency for outbreaks.

Limitations

There were several limitations to this study. First, the small sample size may introduce biases by skewing the data to the perceptions of those with whom we were able to interview. Second, it was difficult to access MSPAS and MAGA staff at the local level, who were involved in this outbreak due to high turnover rates. This information from the local level would have provided an even more fine-tuned understanding of the strengths and drawbacks of the outbreak response. Third, the retrospective nature of this study made it difficult to identify exact dates for calculating 7-1-7 metrics. Without any written documentation or paper trails pertaining to the outbreak, interviewee recall issues may have made it difficult to identify the exact dates of early response completion.

Despite these limitations, this paper provides insight into the application of 7-1-7 benchmarks to a zoonotic disease and the Guatemalan government's public health emergency response system. By strengthening One Health approaches to public health, guaranteeing funding mechanisms for public health emergencies, and upgrading laboratory capacities, the Guatemalan government may be able to enhance its ability to provide a timely response to future outbreaks. Future use of the 7-1-7 framework could include a larger pool of respondents, a longitudinal data design, or real-time data collection in order to provide greater nuance into outbreak preparedness in Guatemala and elsewhere.

Acknowledgements: The authors would like to thank all the study participants; the Central America Regional Office in the CDC for funding and coordinating this project; the Global Surveillance, Laboratory, and Data Systems Branch, the Global Health Security Team in the CDC's Division for Global Health Protection, and the Influenza Division of the National Center for Immunization and Respiratory Diseases for advising on this project.

References

1. Musa O, Salaudeen A, Akanbi A, Bolarinwa O. Risk Factors, Threats And Prevention Of Highly Pathogenic Avian Influenza (HPAI) In African Countries. African Journal of Clinical and Experimental Microbiology. 2009;10(2).
2. Uyeki TM, Milton S, Abdul Hamid C, Reinoso Webb C, Presley SM, Shetty V, et al. Highly Pathogenic Avian Influenza A(H5N1) Virus Infection in a Dairy Farm Worker. New England Journal of Medicine. 2024;390(21):2028-9.
3. Adlhoch C, Fusaro A, Gonzales JL, Kuiken T, Marangon S, Mirinaviciute G, et al. Avian influenza overview December 2022 – March 2023. EFSA Journal. 2023;21(3).
4. Awada L. Update of the Global and regional epidemiological situation of avian influenza outbreaks. World Organisation for Animal Health; 2023.
5. Harvey J, Mullinax J, Runge M, Prosser D. The changing dynamics of highly pathogenic avian influenza H5N1: Next steps for management & science in North America. Biological Conservation. 2023;282.
6. Bochner AF, Makumbi I, Aderinola O, Abayneh A, Jetoh R, Yemanaberhan RL, et al. Implementation of the 7-1-7 target for detection, notification, and response to public health threats in five countries: a retrospective, observational study. The Lancet Global Health. 2023;11(6):e871-e9.
7. Frieden TR, Lee CT, Bochner AF, Buissonnière M, McClelland A. 7-1-7: an organising principle, target, and accountability metric to make the world safer from pandemics. The Lancet. 2021;398(10300):638-40.
8. Why 7-1-7? The evidence behind the global target for containing epidemic threat. In: Alliance --, editor. p. 1-3.
9. Bollyky TJ, Hulland EN, Barber RM, Collins JK, Kiernan S, Moses M, et al. Pandemic preparedness and COVID-19: an exploratory analysis of infection and fatality rates, and contextual factors associated with preparedness in 177 countries, from Jan 1, 2020, to Sept 30, 2021. The Lancet. 2022;399(10334):1489-512.
10. Guidance and tools for conducting an early action review (EAR): rapid performance improvement for outbreak detection and response. Geneva: World Health Organization; 2023.
11. UNICEF. Human-centered approach for health. 2018.
12. Chaney SC, Mechael P. So Many Choices, How Do I Choose? Considerations for Selecting Digital Health Interventions to Support Immunization Confidence and Demand. Journal of Medical Internet Research. 2023;25:e47713.
13. Hopkins KL, Underwood T, Iddrisu I, Woldemeskel H, Bon HB, Brouwers S, et al. Community-Based Approaches to Increase COVID-19 Vaccine Uptake and Demand: Lessons Learned from Four UNICEF-Supported Interventions. Vaccines. 2023;11(7):1180.
14. Ivanova V, Shahabuddin ASM, Sharkey A, Johri M. Advancing Immunization Coverage and Equity: A Structured Synthesis of Pro-Equity Strategies in 61 Gavi-Supported Countries. Vaccines. 2023;11(1):191.
15. Jalloh MF, Patel P, Sutton R, Kulkarni S, Toure M, Wiley K, et al. Qualitative assessment of caregiver experiences when navigating childhood immunisation in urban communities in Sierra Leone. BMJ Open. 2022;12(5):e058203.
16. World Animal Health Information System [Available from: https://www.woah.org/en/what-we-do/animal-health-and-welfare/disease-data-collection/world-animal-health-information-system/.
17. Organization PAH. Epidemiological Update: Outbreaks of avian influenza caused by influenza A(H5N1) in the Region of the Americas. PAHO; 2023.

18. Clara A, Dao ATP, Do TT, Tran PD, Tran QD, Ngu ND, et al. Factors Influencing Community Event-based Surveillance: Lessons Learned from Pilot Implementation in Vietnam. Health Security. 2018;16(S1):S-66-S-75.
19. Technical Guidelines for Integrated Disease Surveillance and Response in the African Region. Brazzaville: World Health Organization. Regional Office for Africa; 2010.
20. Plan De Desarrollo Departamental 2011-2025. Secretaria de Planificacion y Programacion de la Presidencia de Izabal; 2010.
21. WHO information forthe molecular detectionof influenza viruses. The World Health Organization; 2021.
22. Vu T. Epidemics as Politics with Case Studies from Malaysia, Thailand, and Vietnam. Global Health Governance. 2011;IV(2).
23. Ruan LK, Jeffrey; Crete-Nishihata, Masashi. Censored Contagion: How Information on the Coronovirus is Managed on Chinese Social Media. 2020.
24. Shir-Raz Y, Elisha E, Martin B, Ronel N, Guetzkow J. Censorship and Suppression of Covid-19 Heterodoxy: Tactics and Counter-Tactics. Minerva. 2023;61(3):407-33.
25. Stalmeijer RE, McNaughton N, Van Mook WNKA. Using focus groups in medical education research: AMEE Guide No. 91. Medical Teacher. 2014;36(11):923-39.
26.. Kruegar RC, Mary Anne. Designing and Conducting Focus Group Interviews. In: Kruegar rC, Mary Anne; Donner, Jonathan; Kirsch, Stuart; Maack, Jonathan, editor. Social Analysis: Selected Tools and Techniques. Washington D.C.: The World Bank; 2001. p. 4-23.
27. Charlton CL, Hull N, Sloma CR, Bonifas M, Johnson M, Strain AK, Wolford T, Staley S, Perkins C, Razzaque R, Courtney C, Bind EP.2021.How To Prepare for the Unexpected: a Public Health Laboratory Response. Clin Microbiol Rev 34:10.1128/cmr.00183-20.https://doi.org/10.1128/cmr.00183-20
28. Skeels MR. Laboratories and Disease Surveillance. Military Medicine. 2000 Jul 1;165(suppl_2):16–9.
29. McDade JE, Hausler WJ. Modernization of Public Health Laboratories in a Privatization Atmosphere. J Clin Microbiol. 1998 Mar;36(3):609–13.
30. Branda F, Mohapatra RK, Tuglo LS, Ciccozzi M, Scarpa F. Real-time epidemiological surveillance data: tracking the occurrences of avian influenza outbreaks around the world. BMC Res Notes. 2025 Mar 4;18(1):95.
31. Fanelli A, Awada L, Caceres-Soto P, Diaz F, Grillo T, Gizo I, et al. Sensitivity of an international notification system for wildlife diseases: A case study using the OIE-WAHIS data on tularemia. Zoonoses and Public Health. 2022;69(4):286–94.
32 Sazzad HMS, Labrique AB, Teo CG, Luby SP, Gurley ES. Surveillance at Private Laboratories Identifies Small Outbreaks of Hepatitis E in Urban Bangladesh. Am J Trop Med Hyg. 2017 Feb 8;96(2):395–9.
33. Cãceres P, Awada L, Weber-Vintzel, Morales R, Meske M, Tizzani P. The World Animal Health Information System as a tool to support decision-making and research in animal health. Scientific and Technical Review. 2023; 41: 242-251
34. Chien Y-J. How Did International Agencies Perceive the Avian Influenza Problem? The Adoption and Manufacture of the ‘One World, One Health’ Framework. In: Dingwall R, Hoffman L, Staniland K, editors. Pandemics and Emerging Infectious Diseases: The Sociological Agenda :The Sociological Agenda: John Wiley & Sons Ltd; 2013. p. 46-58.
35. Hinjoy S, Wongkumma A, Kongyu S, Smithsuwan P, Suangtho P, Yingyong T, et al. An Assessment of Epidemiology Capacity in a One Health Team at the Provincial Level in Thailand. Veterinary Sciences. 2016;3(4):30.

36. Organization PAH. ONE HEALTH - Action Track 2 [Available from: https://www.paho.org/en/one-health-action-track-2.
37. Innes GK, Lambrou AS, Thumrin P, Thukngamdee Y, Tangwangvivat R, Doungngern P, et al. Enhancing global health security in Thailand: Strengths and challenges of initiating a One Health approach to avian influenza surveillance. One Health. 2022;14.
38. King C-C, Kao C-L, d D-PL, Cheng M-C, Yen H-L, Lee M-S, et al. Seven integrated influenza surveillance systems in Taiwan. International Congress Series. 2001;1219:107-18.
39. Bordier M, Binot TU-AA, Hendrikx P, Goutard FL. Characteristics of One Health surveillance systems: A systematic literature review. Preventive Veterinary Medicine. 2020;181.

supplement 1

Click here to access/download
Supporting Information
SI.docx

Click here to access/download
Supporting Information
SII.docx

Bottleneck and Enabler Evaluation of Avian Influenza Health Event — Guatemala, January-February 2023

Parsa Bastani[1], Edgar Bailey Leonardo[2], Jose Carlos Monzon Fuentes[2], Cesár Conde Pereira[3], Emily Zielinski Gutierrez[2], Parminder S. Suchdev[2*]

1. Epidemic Intelligence Service, Centers for Disease Control and Prevention, Atlanta, GA 30329, USA
2. Central America Office, Division of Global Health Protection, Centers for Disease Control and Prevention, Guatemala City, Guatemala.
3. Ministry of Public Health and Social Assistance, Guatemala

***Corresponding author:**
Parminder S Suchdev, MD, MPH
U.S. Embassy Guatemala
Boulevard Austracio 11-51, Zone 16, Guatemala City, 01016
psuchdev@cdc.gov

Sources of Support: This work was funded by the Centers for Disease Control & Prevention

Additional disclosure: The findings and conclusions in this report are those of the authors and do not necessarily represent the official position of the US Centers for Disease Control and Prevention.

Data Sharing: Data described in the manuscript, code book, and analytic code will be made available upon request.

Author Contributions: PB, EBL, and PSS designed the study. PB designed the semi-structured interview guide and conduct analyses. PB, EBL and JMF conducted interviews. PB and PSS led the manuscript development and are primarily responsible for the final content. All authors have read and approved the final manuscript.

Running title: Evaluation of avian influenza response in Guatemala

Abstract

In February 2023, H5N1 was identified in 11 wild pelicans in Izabal, Guatemala. These were the first known cases of H5N1 in the country. This study assessed the timeliness of the response to this One Health event using the “7-1-7” benchmarks, which propose the following metrics: detection within seven days, notification within one day, and completion of early response within seven days. Open-ended interviews were conducted in September 2023 with nine key informants from the Ministry of Agriculture, Livestock, and Food (MAGA) and the Ministry of Health and Social Assistance (MSPAS) who were directly involved in the response. Participants included epidemiologists, laboratory analysts, and other relevant personnel. Interviews were analyzed using UNICEF’s “Human-Centered Design 4 Health” approach to qualitative fieldwork. Detection and notification were completed in one day, while early response was completed in 34 days. Key enablers of the response included interregional notification and cooperation, availability of earmarked emergency funds, event-based surveillance, and support from laboratories across public and private sectors. Reported bottlenecks included limited national laboratory testing capacity, challenges in inter-agency and intra-agency communication, workforce constraints, and equipment shortages. This outbreak response met the detection and notification criteria but did not achieve the 7-day target for completing early response activities. As one of the few qualitative studies examining avian influenza response in Central America, these findings highlight how strengthening a One Health approach, particularly in communication, workforce, and laboratory capacity, could enhance preparedness for future outbreaks.

Introduction

Avian influenza outbreaks are a persistent global health concern, affecting both animal and human populations. Specifically, highly pathogenic avian influenza (HPAI) is a highly contagious disease that can be fatal in birds (1). Humans with close or prolonged, unprotected exposures are at a greater risk of infection. Human illnesses range from mild to severe, sometimes leading to death (2). In 2022 and 2023, outbreaks of H5N1, a strain of HPAI, were identified in both domestic and wild birds as the virus spread among migratory waterfowl from Canada and the United States to Mexico, Central, and South America(3). The virus spread mainly along the Pacific flyway and resulted in the first occurrence of HPAI in Argentina, Bolivia, Colombia, Costa Rica, Cuba, Ecuador, Guatemala, Honduras, Panama, Peru, Uruguay, and Venezuela(4). The increased number of outbreaks in bird populations across this region raised concerns that the virus is becoming more fit and adept at spreading (5).

In January 2023, an outbreak of H5N1was suspected in 11 brown pelicans (*Pelecanus occidentalis*) in Izabal, Guatemala, a coastal department that abuts the Caribbean Sea. Local fisherman noticed these sick pelicans and alerted a regional epidemiologist stationed in Izabal. In collaboration with partners, H5N1 was confirmed, and the Guatemalan government coordinated several actions to contain and manage the outbreak. While there was no human spillover, these cases of H5N1 in Guatemala underscore the need to strengthen health security measures and ensure adequate public health systems are in place to address potential outbreaks.

Specifically, early disease detection and response are necessary to contain HPAI outbreaks and minimize their potential spread to humans. One tool available in helping countries identify gaps in their preparedness systems and strategies ~~are~~ is the 7-1-7 metrics(6, 7). ~~These~~

ThisareisThis is a set of indicators that measure the timeliness of detection, notification, and early response activities. The goal of 7-1-7 is to ensure governments can meet the following metrics during an outbreak: detect within 7 days of emergence, report to a public health authority responsible for action within 1 day of detection, and complete early response actions within 7 days from reporting to public health authorities. The metric's authors chose these benchmarks based on a detailed examination of past outbreak timeliness data and the World Health Organization's Integrated Disease Surveillance and Response (IDSR) Technical Guidelines (3rd Edition)(7, 8). This approach supplements helps rectify the shortcomings of existing pandemic preparedness measures, which often fail to capture how emergency response systems function in real-time. For example, scores on the WHO Joint External Evaluation (JEE) and Global Health Security Index were not correlated with COVID-19 outcomes, such as standardized infection rates (9). To address this gap, in August 2023, the WHO incorporated 7-1-7 targets into its early action review (EAR) guidance to help countries evaluate the timeliness of their early detection and response activities(10).

A qualitative understanding of issues that serve as bottlenecks and enablers for timely outbreak response is key to achieving 7-1-7 targets. We define these targets as the following: detection was when local residents detected the aberrant health event of sick pelicans along the coast; notification was when the event was reported to the Guatemalan Ministry of Agriculture, Livestock and Food; and early response completion was when the investigation team was deployed, initial risk assessments were completed, laboratory confirmation of the outbreak etiology was obtained, and public health measures, risk communication, and community engagement measures were initiated by human and animal health sectors. This information can help governments direct where to channel funding and resources for improving their outbreak

preparedness. Thus far, one retrospective study has been published on identifying bottlenecks and enablers to 7-1-7 system performance (6). In this study, data collection appeared to primarily take took place through reviewing situations, intra-action and after-action reviews, discussions with subnational and national-level responders, and a 1-day workshop. Given the limited research on 7-1-7 bottlenecks and enablers, additional evaluation tools are needed for evaluating outbreak situations with limited written records that use the 7-1-7 framework..

This study expands upon extant 7-1-7 literature by focusing on a country in Central America, examining an animal outbreak of a zoonotic disease prior to a potential spill-over event, and proposing a unique, simple, and rapid qualitative methodology for collecting and analyzing data on health emergency preparedness. Specifically, this study systematizes the collection of qualitative materials through semi-structured interviews with key officials. Our aims were twofold: 1) to calculate 7-1-7 timeliness metrics during the January H5N1 outbreak in Guatemala, and 2) to identify bottlenecks and enablers that affected this outbreak response.

Methods

Study design and data collection

Recruitment and interviews were conducted between 17/09/2023 and 30/09/2023 with nine key informants in Guatemala involved in the H5N1 response using a purposive sampling strategy. Key informants were selected from government agencies that were most involved in the outbreak. These included epidemiologists, laboratory analysts, and program officers from the Guatemalan Ministry of Agriculture, Livestock and Food (MAGA) and the Ministry of Public Health and Social Assistance (MSPAS). Due to high staff turnover, it was not possible to locate and consent further respondents within the recruitment time frame. Interviews were conducted in

Spanish and English and lasted between 30-60 minutes. Interviews were either conductedconducted either on the phone or in-person in a private location in an office space by PB (an epidemiologist and medical anthropologist) and ELB (an epidemiologist and veterinarian). All respondents were verbally consented prior to each interview and consent was documented in the written field notes. Semi-structured interview guides were administered to each respondent (see Supplement I). Questions covered the research participant's experience and perceptions of the outbreak response during the detection, notification, and response Eachstages. Each interview was recorded and transcribed.

Data analysis

Analysis of interview data was conducted using the rapid inquiry approach published in United Nations International Children's Emergency Fund's (UNICEF) "Human-Centered Design 4 Health."(11) This toolkit was originally designed to reduce the technical barriers for conducting qualitative research on basic health services and has been used in several published studies(12-15). In other words, UNICEF designed this toolkit so that people with no technical training could perform qualitative research in their communities.

We drew on the toolkit's analyses framework using its ""Record Field Research"" and ""Synthesis Tools"" worksheets to identify important themes from the transcripts. We followed UNICEF's overall analysis process: first, we used filled out one ""Record Field Research"" worksheet per interview to analyze each of the interviews and second, we filled out one "Synthesis Tool" per interview subgroup. The first step This comprised of listing the following for each interview: five meaningful quotes, five surprising moments, five suggestions from the interviewer, and five interventions the analyzer (PB and EBL) identified based on the interview

(see supplement II). Second, we grouped placed together the 'Record Field Research' worksheets into subgroups based on the relevant backgrounds of respondents. In this case, we grouped interviews togetherAccordingly, we created four subgroups: 1) national level MAGA staff; 2) local level MAGA staff; 3) national level MSPAS staff, and 4) local level MSPAS staff. based on their respective agency and the level in which they worked (i.e. national or local). Last, we completed one "'Synthesis Tool"' worksheet for each subgroup reviewed the completedby reviewing the ''Record Field Research' worksheets for that each subgroup. This comprised of listing the following per subgroup: three powerful quotes, three things that worked well, three suggestions, three specific issues that need to be addressed, and the subgroup's perception of the overall detection, notification, and response to the outbreak (see supplement II). In the UNICEF methodology, the completion of this "'Synthesis Tool"' worksheet constitutes the analysis process, which equates to the analysis of codes in more conventional qualitative methodologies. in order to fill out one 'Synthesis Tool' worksheet per that subgroup.

However, weThe information captured in the tools we used were also adapted from thethe UNICEF HCD Human Centered Design (HCD) Toolkit to better capture data on disease outbreaks. We specifically changed the "“Synthesis Tool"” worksheet by: replacing references to "immunization services" to "overall outbreak response capacities", collecting three of the most powerful quotes (rather than "3 AHA! Moments") and removing the section on identifying "2 Things We Must Address" to instead highlight the overall group's perspective on detection, notification, and responsee. Even though the primary author is a trained qualitative researcher, the research team strategically chose to use the UNICEF toolkit adaptation in order to build local capacity for qualitative analysis. The goal was to not only train local CDC staff in qualitative

methods, but also to provide a clear, tested, and simple qualitative analysis tool to other CDC field offices seeking to carryout 7-1-7 analyses.

Ethics

This activity was reviewed by the US Centers for Disease Control and Prevention (CDC) and was conducted consistent with applicable federal law and CDC policy. (§See e.g., 45 C.F.R. part 46, 21 C.F.R. part 56; 42 U.S.C. §241(d); 5 U.S.C. §552a; 44 U.S.C. §3501 et seq)

Formatted: Line spacing: Double

Formatted: Font: 12 pt

Results

Assessing timeliness

Detection and notification met 7-1-7 timeliness benchmarks; however, the early response did not meet these benchmarks. Detection and notification both took one day. Our records (**Table 1**) indicate that fisherman from Izabal alerted a regional-level epidemiologist from MAGA of 11 sick pelicans on January 25th, 2023, the same day they noticed the pelicans. The epidemiologist arrived at the scene of the health event on January 26th, 2023, and notified that same day the national office of MAGA, the entity responsible for action. In contrast to the timeliness of detection and notification, the early response took approximately 34 days. On January 27th, MAGA sent officials to bring two deceased pelicans from Izabal to Guatemala City for HPAI testing. Immediately after transferring samples, active surveillance and biosafety and biosecurity measures were put in place including animal quarantine measures and decontamination. Once in the custody of MAGA in Guatemala City, samples were sent to

National Veterinary Services Laboratories (NVSL) in Ames, Iowa for testing. These samples were received by NVSL on February 6th. The H5N1 confirmation from the NVSL in Ames, Iowa was received on February 6th. The delay in the early response was a result of the inability to coordinate a meeting between the MSPAS and Izabal ~~stakeholders~~ community members about protocols for dealing with human transmission. This is a necessary step in the early response because community members should be made aware of how to identify and respond to H5N1 human spillover as soon as possible. While the exact date could not be confirmed, interviews corroborated that this meeting took place, at the earliest, on March 1st.

~~**Table 1: Timeline of significant events in the avian influenza outbreak.**~~

**Timeline**
January 25th: Regional MAGA epidemiologist receives alert from community of 11 sick pelicans
January 26th: Regional MAGA epidemiologist investigates outbreak and alerts national MAGA
January 27th: MAGA sends officials to bring 2 whole pelicans from Izabal to Guatemala City for testing.
January 28th: Private lab in Guatemala confirms H5 in pelican sample
January 30: Samples of Pelicans sent to National Veterinary Services Laboratories (NVSL) in Ames, Iowa
February 6th: Results received from NVSL of samples positive for H5N1
March 1st: Approximate date for meeting between Ministry of Public Health and Social Assistance (MSPAS) and Izabal stakeholders.

Table 1: Timeline of significant events in the avian influenza outbreak.

**Timeline**			
**Response Stage**	**Date**	**Response Stage**	**Activity**
**Detection**	January 25th	**Detection**	Regional MAGA epidemiologist receives alert from community of 11 sick pelicans

Formatted: Font: 11 pt

Formatted: Space After: 8 pt, Line spacing: Multiple 1.08 li

Formatted: Centered

Formatted Table

Formatted: Line spacing: single

**Notification**	January 26th	**Notification**	Regional MAGA epidemiologist investigates outbreak and alerts national MAGA
**Early Response**	January 27th	**Early Response**	MAGA sends officials to bring 2 whole pelicans from Izabal to Guatemala City for testing
	January 28th		Private lab in Guatemala confirms H5 in pelican sample
	January 30th		Samples of Pelicans sent to National Veterinary Services Laboratories (NVSL) in Ames, Iowa
	February 6th		Results received from NVSL of samples positive for H5N1
	March 1st		Approximate date for meeting between Ministry of Public Health and Social Assistance (MSPAS) and Izabal stakeholders

Formatted: Line spacing: single

Formatted: Font: 10 pt, Not Bold

Formatted: Line spacing: single

Formatted: Font: 10 pt, Not Bold

Formatted: Line spacing: single

Formatted: Font: 10 pt, Not Bold

Formatted: Superscript

Formatted: Line spacing: single

Formatted: Line spacing: single

Formatted: Superscript

Formatted: Line spacing: single

Formatted: Font: 10 pt, Not Bold

Enablers

Interregional notification and cooperation

Notifications of avian influenza in neighboring countries from the World Animal Health Information System (WAHIS) placed Guatemalan national authorities on high alert for potential HPAI cases in animals. Launched by the World Organization for Animal Health (WOAH), WAHIS is an early warning and monitoring system that disseminates information about animal health and sends alerts on animal diseases and events in real-time (16) . As one official from MAGA stated,

> "Since November, we were expecting to have the first case. The outbreak was not a big surprise, it was part of surveillance. How we managed the case, it was okay.”

The respondent is referencing the multiple HPAI outbreaks in animals across Latin and South America between October 2022 and January 2023(17). This knowledge helped the Guatemalan government prepare for a potential outbreak.

Emergency funding

For this health event, there was emergency funding available to carry out epidemiological activities. The funds came from the International Regional Organization for Plant and Animal Health (OIRSA), an organization that aims to develop and coordinate programs for the prevention, control, and eradication of diseases and pests in its member countries (Belize, Costa Rica, Dominican Republic, El Salvador, Guatemala, Honduras, Mexico, Nicaragua, and Panama). The emergency funds from OIRSA are $100,000 per year, and if an outbreak needs additional support, the Regional International Council of Ministries of Agriculture (CIRSA) (OIRSA´s highest authority), can allocate an additional $1 million toward a response. For this emergency, MAGA drew on OIRSA to obtain funds for gasoline, vehicles, per diems, meals, and overnight stays for field teams. The availability of OIRSA funds allowed MAGA to respond to health events without needing to submit requests and obtain approvals for other internal funds. These emergency funds, however, have depleted since the conclusion of this health event. Respondents echoed a call for "for the permanent appropriation of emergency funds within their nationally approved budgets.

Community event-based surveillance (CEBS)

Community event-based surveillance (CEBS) was pivotal to the Guatemalan~~'s~~ government's detection of H5N1 in pelicans. CEBS is a public health strategy in which

community members participate in detecting unusual health events or behaviors (18). Typically, this involves trained surveillance informants identifying and reporting events in the community that have public health significance (19). In Guatemala, community surveillance was developed empirically through past experiences with disease control strategies, such as the eradication of swine fever (2011-2015) and the management of low-pathogenic avian influenza since the 2000s. During these periods, MAGA personnel identified community leaders to establish a passive surveillance network. As a result, communities know whom to contact and where to report disease suspicions or outbreaks, which facilitated early detection in cases. In Izabal, CEBS was enabled by the strong relationship between community members and the regional level MAGA epidemiological team. As one respondent stated,

> "There is a very good communication between these people [fisherman] and the district epidemiologist. Do you know why? We give vaccines and some medicines. They are very grateful, so any action or thing that happens, they call us. We have a few areas where this communication is hard, the areas where we have more indigenous people. [The indigenous people think that] once you call MAGA, they come to kill [their] animals."

The relationship between the community and government was enabled in part by the demographic and linguistic composition of Izabal of Spanish speaking, primarily non-Indigenous populations. In contrast to other parts of the country, with higher percentages of indigenous populations, government institutions are more easily able to build trust and communicate with the local population in Izabal. According to a 2010 report, Izabal is 25.56 % indigenous(20). In the Puerto Barrios municipality of Izabal, where the pelicans were found, only 3% of the population is indigenous(20). During this outbreak, fisherman from Izabal directly called the

regional epidemiologist to inform that person about the 11 dying pelicans. The point of contact for the local fisherman was a MAGA epidemiologist who had established a strong network of contacts after working in the area for 12 years.

Wide network of laboratories

The government's ability to collaborate with private laboratories enabled a timely response. The pelican samples were brought from Izabal to Guatemala City on the evening of Friday January 27th. At this time, the national MAGA laboratories were closed for the weekend and the epidemiological team instead took the samples to Laboratorio Sistemas y Equipos, a private laboratory. At the time, there were 4 veterinary diagnostic laboratories that the Guatemalan government had certified and provided official licenses. A polymerase chain reaction (real-time PCR) test revealed that the samples were positive for Avian Influenza H5 on January 28th, 2023, which was the second day of initiating an early response.

Bottlenecks

National Testing Capacity

Governmental and private laboratories had the reagents to test for H5 but not the N1 subtype in Influenza virus type A (21). Without the adequate diagnostic equipment to confirm the subtype H5N1 within Guatemala, the state's public health system was reliant on reference laboratories in the United States. The samples (on one Flinders Technology Associates® card with the virus isolated) were shipped on Monday January 30th to NVSL in Ames, Iowa, five days after the initial notification on Wednesday, January 25th. MAGA received notification from NSVL on February 6th 2023 that the samples were positive for H5N1. The lag between shipment

and notification was due to severe weather conditions in the USA that impacted the courier service.

Workforce Need

Workforce needs were noted by all respondents. For example, a respondent from the Department of Surveillance and Control of the Directorate of Epidemiology and Risk Management in MSPAS stated,

> "the main problem is that there are no human resources, it is the same person in charge of surveillance, the same person who goes to the response, the same person to capacity build the team, the same person to be in the field, it's a limitation to our resources in general"

As pointed out, the same national-level epidemiologist who oversees surveillance ~~are~~ is also in charge of outbreak responses for all respiratory diseases. The lack of adequate, non-emergency funding to bolster staffing slowed down the responsiveness of MSPAS.

PPE Equipment:

This health event highlighted that the workforce lacked equipment and training for addressing pathogenic and contagious diseases in animals. An interviewee from MAGA's national office was unsure if response teams knew how to don and doff PPE and to safely take and send biological samples. These concerns were echoed at the regional level. One respondent who worked on the ground in Izabal during this health event stated:

> "The truth is that we were not prepared because we did not know what we were facing. The Programa Nacional de Sanidad Avícola (PROSA) response team [that was sent from the national level] had its protective equipment, but it did not have protection for all individuals

[involved in the response who lived in the Izabal region]. [Our regional team] didn't have swabs, we didn't even have coolers [to transport the dead pelicans], something so simple...we weren't prepared, the truth is that the team wasn't prepared for what was coming."

After voicing these concerns to colleagues at the national level, this respondent was promised additional supplies but was sent 5 pairs of gloves, a bag of straws, and four overalls to address further surveillance and biosecurity response efforts due to resource constraints. Early in the response, the respondent had to reuse gloves while handling the deceased pelicans because they had already run out of clean gloves.

Intra-agency and inter-agency communication and planning

During H5N1 outbreaks, human health sectors should be involved in the response to ensure human spillover can be contained. During this health event, human health sector involvement was the largest barrier to early response completion. One MSPAS official blamed theThere was a lack of timelyclear communication between animal and human healthnotification from MAGA as the primary reason for the delay in action sectors in Guatemala. The completion of the early response was most impacted by slow coordination between MSPAS and MAGA. MSPAS officials blamed their lack of early response on a delay in notification from MAGA. As this officialone official recounted,

"One of the issues we complained about is that the new information did not come to us in a timely way…for this reason the response happened a bit late. We would have wanted to know earlier…we wanted the information when they had the suspicion."

From information obtained from interviews, it appears that MSPAS received this notification between 5 and 7 days after the initial notification to MAGA.

However, our findings revealindicate that internal agency communication and planning within MSPAS appeared to be even more key in delaying the response played even a bigger role in the delayed response. Although MSPAS received the notification, they did not take any on-the-ground action until at least March 1st, when a meeting was held with community members and officials in IzabalOnce MSPAS received the notification, they did not take any on-the-ground action until at least March 1st, the day when MSPAS held a meeting with community members and officials in Izabal. Due to insufficient protocols and processespreparadness for One Health emergency situations, Iit took MSPAS several weeks to to mobilize the necessary personnel, equipment, and fundinglocatefind the personnel, equipment, and funding. In short, MSPAS was under-resourced and unprepared to respond effectively to the health event. Thus, even with the initial delay in notification from MAGA, there appeared to be significant gaps in internal coordination and limited capacity within MSPAS to initiate a timely, cross-sectoral response aimed at preventing potential disease spillover to humans..In other words, MSPAS was under-resourced and unprepared to respond to the health event. Thus, even with the delay in notification from MAGA, there appeared to be unclear lines of communication within MSPAS to did not have the capacity to initiate a cross-sectoral response that would protect humans against potential disease spillover.

The regional level epidemiological team of MAGA reported that they did not have a clear understanding of their role for both routine surveillance and outbreak response vis-a-vis their counterparts at the national level. Although this did not impact the timeliness of initiating a response, communication issues affected the quality of the response. For this health event, the regional level team worked with the PROSA, a subunit of MAGA that led response efforts. Our respondent at the regional level did not know if PROSA's domain of work included more routine

surveillance or if it was limited to responses to health events and outbreaks. When discussing PROSA's response activities, the respondent stated,

> "The next week, another group [from PROSA] came, but no one gave them the information from last week and there was no one who served as their leader. [The week after that], another group [from PROSA] came, but no one gave them the information about the previous week"

This respondent pointed to a disjointed response from PROSA and noted that the area map they were working was outdated. On it were communities that no longer existed or had moved. Not having consulted the regional epidemiological team of MAGA, the early response may have encountered delays, duplications, and a lack of coverage.

Discussion

Timeliness Achievements

Our results indicated that detection and notification met the targets of seven and one days, respectively. However, the completion of the early response far exceeded the 7-day benchmark and instead took approximately 34 days (**Tables 1 and 2**). All steps pertaining to the animal health sector were completed in a timely manner, including deploying investigation/response teams, conducting epidemiological analysis and an initial risk assessment, obtaining laboratory confirmation of the outbreak etiology, initiating risk communication in affected communities, and establishing a coordination mechanism. The delays in meeting the benchmark were due to –issues coordinating with the human health sector (MSPAS), which took 34 days from the date of notification. If there had been human spillover during this outbreak, containment efforts could have been severely hampered as a result.

Table 2: 7-1-7 Milestone Dates

7-1-7 Milestone Dates	Date	Timeliness (in days)	Target (in Days)	Met Target (Yes/No)
Emergence	25 January 2023	Not Applicable	Not Applicable	Not Applicable
Detection	25 January 2023	0	7	Yes
Notification	26 January 2023	1	1	Yes
Early Response Action Completion	1 March 2023	34	7	No

Recommendations

This study highlighted two obstacles in carrying out 7-1-7 assessments. First, bottleneck and enabler analysis could benefit from additional qualitative methods ~~more expansive discussion of methods~~. The previously published study suggests that investigators review documentation of outbreak response efforts and conduct workshops to gather consensus around bottlenecks and enablers(6). As was the case in our study, there was little publicly available documentation on the outbreak response. For this reason, we relied almost entirely on qualitatively collected data. We chose to focus data collection on interviews due to the ethical challenges of carrying out workshops. Workshops would function similarly to focus groups, a qualitative method in which a group of people are asked to discuss and collectively answer questions posed by a moderator. The problem with avian influenza outbreaks, like many other disease outbreaks, is that they are highly politicized health events (22-24). There are many powerful players placed at risk during an outbreak, such as tourism industries, livestock companies, and politicians. Given the sensitive climate surrounding outbreaks, we decided that focus groups might hinder an honest dialogue between government stakeholders about bottlenecks. This decision was supported by the literature, which notes that focus groups are not effective if participants fear future reprisal or negative impacts because of their honesty (25, 26).

In addition to these suggestions about conducting 7-1-7 assessments, this study's qualitative methodology provides unique insight into ~~specific~~ the enablers and roadblocks~~areas~~

Formatted: Indent: First line: 0.5"

~~of~~to avian influenza disease surveillance and preparedness in Guatemala.~~ that may serve as roadblocks in future outbreaks~~. This analysis confirmed that multinational disease reporting systems (e.g. WAHIS), CEBS, and private-public lab partnerships can enable efficient outbreak detection (27-33). In contrast, ~~Corroborated by the 7-1-7 bottleneck literature~~, this study also corroborated literature that faltering cooperation between the human and animal health sectors ~~in this avian influenza outbreak was a primary reason for a~~can delay ~~in~~ early response activities in outbreak settings(6). In the realm of influenza, this separation of animal and human surveillance is quite common (27, 28). In line with calls from the Pan American Health Organization, evidence from this research suggests that the risks of emerging zoonotic epidemics could be mitigated if all member states (including Guatemala) further enhance coordinated surveillance across the human-animal-environmental interface (29). This One Health approach could more efficiently facilitate coordinated communication, data collection and transfer, and resource allocation.

There are many examples of well-coordinated One Health surveillance approaches in the literature. In Thailand, a One Health surveillance system was coordinated by a single office (the Coordinating Unit for One Health), which had the task of maintaining interagency communication and presiding over the cross-governmental implementation of One Health activities (30). Taiwan also implements a One Health approach, whereby it integrates wild bird virologic surveillance, domestic avian influenza surveillance, market avian virologic surveillance, swine influenza surveillance, sentinel physician surveillance, human virologic surveillance, and severe case surveillance (31). These and other models (32) may serve as important starting points for designing a One Health surveillance and outbreak response system.

Efforts to develop a One Health response have been underway in Guatemala. Since 2021, the Centers for Disease Control and Prevention (CDC) have been promoting the One Health approach in Central America, including Guatemala. CDC engaged with the Ministry of Public Health and Social Assistance (MSPAS), the Ministry of Agriculture, Livestock, and Food (MAGA), and the Ministry of Environment and Natural Resources (MARN) to integrate efforts. If the governmental agreement is signed, the One Health Technical Working Group could have an official mandate that facilitates its operation and cooperation among the ministries. This could enable the establishment of more concrete and effective actions for the detection, notification, and response to zoonotic diseases, thereby improving public health in Guatemala. Institutionalization could also strengthen coordination and communication between the human, animal, and environmental health sectors, promoting a faster and more efficient response to health emergencies.

Last, Guatemalan outbreak response capacities could benefit from strengthening diagnostic testing capabilities and workforce. During the health event in January 2023, the national MAGA laboratories did not have the capacity to test for H5N1 and relied on the Pan American Health Organization and the Centers for Disease Control and Prevention to test samples in laboratories outside of Guatemala. Following this incident, gaps in testing capacity were identified in the national animal health laboratory capacities, and improvements were made. Strengthening MSPAS's capacity to test for HPAI would improve 7-1-7 metrics and further prepare the agency for outbreaks.

Limitations

There were several limitations to this study. First, the small sample size may introduce biases by skewing the data to the perceptions of those with whom we were able to interview. Second, it was difficult to access MSPAS and MAGA staff at the local level, who were involved in this outbreak due to high turnover rates. This information from the local level would have provided an even more fine-tuned understanding of the strengths and drawbacks of the outbreak response. Third, the retrospective nature of this study made it difficult to identify exact dates for calculating 7-1-7 metrics. ~~These~~ Without any written documentation or paper trails pertaining to the outbreak, interviewee recall issues may have made it difficult to identify the exact dates of early response completion.

Despite these limitations, this paper provides insight into the application of 7-1-7 benchmarks to a zoonotic disease and the Guatemalan government's public health emergency response system. By strengthening One Health approaches to public health, guaranteeing funding mechanisms for public health emergencies, and upgrading laboratory capacities, the Guatemalan government may be able to enhance its ability to provide a timely response to future outbreaks. Future use of the 7-1-7 framework could include a larger pool of respondents, a longitudinal data design, or real-time data collection in order to provide greater nuance into outbreak preparedness in Guatemala and elsewhere.

Acknowledgements: The authors would like to thank all the study participants; the Central America Regional Office in the CDC for funding and coordinating this project; the Global Surveillance, Laboratory, and Data Systems Branch, the Global Health Security Team in the CDC's Division for Global Health Protection, and the Influenza Division of the National Center for Immunization and Respiratory Diseases for advising on this project.

References

1. Musa O, Salaudeen A, Akanbi A, Bolarinwa O. Risk Factors, Threats And Prevention Of Highly Pathogenic Avian Influenza (HPAI) In African Countries. African Journal of Clinical and Experimental Microbiology. 2009;10(2).
2. Uyeki TM, Milton S, Abdul Hamid C, Reinoso Webb C, Presley SM, Shetty V, et al. Highly Pathogenic Avian Influenza A(H5N1) Virus Infection in a Dairy Farm Worker. New England Journal of Medicine. 2024;390(21):2028-9.
3. Adlhoch C, Fusaro A, Gonzales JL, Kuiken T, Marangon S, Mirinaviciute G, et al. Avian influenza overview December 2022 – March 2023. EFSA Journal. 2023;21(3).
4. Awada L. Update of the Global and regional epidemiological situation of avian influenza outbreaks. World Organisation for Animal Health; 2023.
5. Harvey J, Mullinax J, Runge M, Prosser D. The changing dynamics of highly pathogenic avian influenza H5N1: Next steps for management & science in North America. Biological Conservation. 2023;282.
6. Bochner AF, Makumbi I, Aderinola O, Abayneh A, Jetoh R, Yemanaberhan RL, et al. Implementation of the 7-1-7 target for detection, notification, and response to public health threats in five countries: a retrospective, observational study. The Lancet Global Health. 2023;11(6):e871-e9.
7. Frieden TR, Lee CT, Bochner AF, Buissonnière M, McClelland A. 7-1-7: an organising principle, target, and accountability metric to make the world safer from pandemics. The Lancet. 2021;398(10300):638-40.
8. Why 7-1-7? The evidence behind the global target for containing epidemic threat. In: Alliance --, editor. p. 1-3.
9. Bollyky TJ, Hulland EN, Barber RM, Collins JK, Kiernan S, Moses M, et al. Pandemic preparedness and COVID-19: an exploratory analysis of infection and fatality rates, and contextual factors associated with preparedness in 177 countries, from Jan 1, 2020, to Sept 30, 2021. The Lancet. 2022;399(10334):1489-512.
10. Guidance and tools for conducting an early action review (EAR): rapid performance improvement for outbreak detection and response. Geneva: World Health Organization; 2023.
11. UNICEF. Human-centered approach for health. 2018.
12. Chaney SC, Mechael P. So Many Choices, How Do I Choose? Considerations for Selecting Digital Health Interventions to Support Immunization Confidence and Demand. Journal of Medical Internet Research. 2023;25:e47713.
13. Hopkins KL, Underwood T, Iddrisu I, Woldemeskel H, Bon HB, Brouwers S, et al. Community-Based Approaches to Increase COVID-19 Vaccine Uptake and Demand: Lessons Learned from Four UNICEF-Supported Interventions. Vaccines. 2023;11(7):1180.
14. Ivanova V, Shahabuddin ASM, Sharkey A, Johri M. Advancing Immunization Coverage and Equity: A Structured Synthesis of Pro-Equity Strategies in 61 Gavi-Supported Countries. Vaccines. 2023;11(1):191.
15. Jalloh MF, Patel P, Sutton R, Kulkarni S, Toure M, Wiley K, et al. Qualitative assessment of caregiver experiences when navigating childhood immunisation in urban communities in Sierra Leone. BMJ Open. 2022;12(5):e058203.
16. World Animal Health Information System [Available from: https://www.woah.org/en/what-we-do/animal-health-and-welfare/disease-data-collection/world-animal-health-information-system/.
17. Organization PAH. Epidemiological Update: Outbreaks of avian influenza caused by influenza A(H5N1) in the Region of the Americas. PAHO; 2023.

18. Clara A, Dao ATP, Do TT, Tran PD, Tran QD, Ngu ND, et al. Factors Influencing Community Event-based Surveillance: Lessons Learned from Pilot Implementation in Vietnam. Health Security. 2018;16(S1):S-66-S-75.
19. Technical Guidelines for Integrated Disease Surveillance and Response in the African Region. Brazzaville: World Health Organization. Regional Office for Africa; 2010.
20. Plan De Desarrollo Departamental 2011-2025. Secretaria de Planificacion y Programacion de la Presidencia de Izabal; 2010.
21. WHO information forthe molecular detectionof influenza viruses. The World Health Organization; 2021.
22. Vu T. Epidemics as Politics with Case Studies from Malaysia, Thailand, and Vietnam. Global Health Governance. 2011;IV(2).
23. Ruan LK, Jeffrey; Crete-Nishihata, Masashi. Censored Contagion: How Information on the Coronovirus is Managed on Chinese Social Media. 2020.
24. Shir-Raz Y, Elisha E, Martin B, Ronel N, Guetzkow J. Censorship and Suppression of Covid-19 Heterodoxy: Tactics and Counter-Tactics. Minerva. 2023;61(3):407-33.
25. Stalmeijer RE, McNaughton N, Van Mook WNKA. Using focus groups in medical education research: AMEE Guide No. 91. Medical Teacher. 2014;36(11):923-39.
26.
~~26~~. Kruegar RC, Mary Anne. Designing and Conducting Focus Group Interviews. In: Kruegar rC, Mary Anne; Donner, Jonathan; Kirsch, Stuart; Maack, Jonathan, editor. Social Analysis: Selected Tools and Techniques. Washington D.C.: The World Bank; 2001. p. 4-23.
27. Charlton CL, Hull N, Sloma CR, Bonifas M, Johnson M, Strain AK, Wolford T, Staley S, Perkins C, Razzaque R, Courtney C, Bind EP.2021.How To Prepare for the Unexpected: a Public Health Laboratory Response. Clin Microbiol Rev 34:10.1128/cmr.00183-20.https://doi.org/10.1128/cmr.00183-20
28. Skeels MR. Laboratories and Disease Surveillance. Military Medicine. 2000 Jul 1;165(suppl_2):16–9.
29. McDade JE, Hausler WJ. Modernization of Public Health Laboratories in a Privatization Atmosphere. J Clin Microbiol. 1998 Mar;36(3):609–13.
30. Branda F, Mohapatra RK, Tuglo LS, Ciccozzi M, Scarpa F. Real-time epidemiological surveillance data: tracking the occurrences of avian influenza outbreaks around the world. BMC Res Notes. 2025 Mar 4;18(1):95.
31. Fanelli A, Awada L, Caceres-Soto P, Diaz F, Grillo T, Gizo I, et al. Sensitivity of an international notification system for wildlife diseases: A case study using the OIE-WAHIS data on tularemia. Zoonoses and Public Health. 2022;69(4):286–94.
32 Sazzad HMS, Labrique AB, Teo CG, Luby SP, Gurley ES. Surveillance at Private Laboratories Identifies Small Outbreaks of Hepatitis E in Urban Bangladesh. Am J Trop Med Hyg. 2017 Feb 8;96(2):395–9.
33. Cãceres P, Awada L, Weber-Vintzel, Morales R, Meske M, Tizzani P. The World Animal Health Information System as a tool to support decision-making and research in animal health. Scientific and Technical Review. 2023; 41: 242-251

34~~27~~. Chien Y-J. How Did International Agencies Perceive the Avian Influenza Problem? The Adoption and Manufacture of the 'One World, One Health' Framework. In: Dingwall R, Hoffman L, Staniland K, editors. Pandemics and Emerging Infectious Diseases: The Sociological Agenda :The Sociological Agenda: John Wiley & Sons Ltd; 2013. p. 46-58.

Formatted: Normal

3528. Hinjoy S, Wongkumma A, Kongyu S, Smithsuwan P, Suangtho P, Yingyong T, et al. An Assessment of Epidemiology Capacity in a One Health Team at the Provincial Level in Thailand. Veterinary Sciences. 2016;3(4):30.
3629. Organization PAH. ONE HEALTH - Action Track 2 [Available from: https://www.paho.org/en/one-health-action-track-2.
3037. Innes GK, Lambrou AS, Thumrin P, Thukngamdee Y, Tangwangvivat R, Doungngern P, et al. Enhancing global health security in Thailand: Strengths and challenges of initiating a One Health approach to avian influenza surveillance. One Health. 2022;14.
3138. King C-C, Kao C-L, d D-PL, Cheng M-C, Yen H-L, Lee M-S, et al. Seven integrated influenza surveillance systems in Taiwan. International Congress Series. 2001;1219:107-18.
3239. Bordier M, Binot TU-AA, Hendrikx P, Goutard FL. Characteristics of One Health surveillance systems: A systematic literature review. Preventive Veterinary Medicine. 2020;181.

Formatted: French (France)

Thank you for the thoughtful and detailed review of our manuscript. We have carefully revised and responded to each point raised by the reviewers and academic editor below in red font. We have also submitted a revised manuscript in track changes, as well as an unmarked revised manuscript.

In reference to some of the high-level issues highlighted by the Academic Editor, we have made the recommended changes to the table and discussion.

Editor comments:

1. Please include a complete copy of PLOS' questionnaire on inclusivity in global research in your revised manuscript. Our policy for research in this area aims to improve transparency in the reporting of research performed outside of researchers' own country or community. The policy applies to researchers who have travelled to a different country to conduct research, research with Indigenous populations or their lands, and research on cultural artefacts. The questionnaire can also be requested at the journal's discretion for any other submissions, even if these conditions are not met. Please find more information on the policy and a link to download a blank copy of the questionnaire here: https://journals.plos.org/globalpublichealth/s/best-practices-in-research-reporting. Please upload a completed version of your questionnaire as Supporting Information when you resubmit your manuscript.
    - Done
2. We do not publish any copyright or trademark symbols that usually accompany proprietary names, eg (R), (C), or TM (e.g. next to drug or reagent names). Please remove all instances of trademark/copyright symbols throughout the text, including ® on page 15.
    - Done

3. We note that your Data Availability Statement is currently as follows: [All relevant data are within the paper.]

    Please confirm at this time whether or not your submission contains all raw data required to replicate the results of your study. Authors must share the "minimal data set" for their submission. PLOS defines the minimal data set to consist of the data required to replicate all study findings reported in the article, as well as related metadata and methods (https://journals.plos.org/plosone/s/data-availability#loc-minimal-data-set-definition).

    For example, authors should submit the following data:
    - The values behind the means, standard deviations and other measures reported;
    - The values used to build graphs;
    - The points extracted from images for analysis.

    Authors do not need to submit their entire data set if only a portion of the data was used in the reported study.
    If your submission does not contain these data, please either upload them as Supporting Information files or deposit them to a stable, public repository and provide us with the relevant URLs, DOIs, or accession numbers. For a list of recommended repositories, please

see https://journals.plos.org/plosone/s/recommended-repositories.

If there are ethical or legal restrictions on sharing a de-identified data set, please explain them in detail (e.g., data contain potentially sensitive information, data are owned by a third-party organization, etc.) and who has imposed them (e.g., an ethics committee). Please also provide contact information for a data access committee, ethics committee, or other institutional body to which data requests may be sent. If data are owned by a third party, please indicate how others may request data access.

- **Confirmed**

Reviewer #1:

The manuscript is well written and presents a valuable and timely contribution to One Health area. The content is easy to comprehend and the data is relevant. However, the manuscript would benefit from a comprehensive revision, particularly with methodology and data reporting. It would also be great to see further more elaboration on causes/reasons of no 'on-the-ground' action by MSPAS (shared paragraph below). This no-action delayed the effective response even when they managed to adhere to 7-1-7 for detection and investigation initiation phases with limited resource constraint.

"However, internal agency communication and planning in MSPAS appeared to be even more key in delaying the response. Once MSPAS received the notification, they did not take any on-the-ground action until at least March 1st. It took MSPAS several weeks to find the personnel, equipment, and funding. Thus, even with the delay in notification from MAGA, there appeared to be unclear lines of communication within MSPAS to initiate a cross-sectoral response that would protect humans against potential disease spillover."

We thank you for these comments and constructive feedback. We appreciate your recognition of the manuscript's contribution to the One Health field and your comments on its clarity and relevance. In response to your suggestion regarding elaboration on the lack of 'on-the-ground' action by MSPAS, we have revised the paragraph to more clearly articulate that the delay stemmed from internal challenges, including limited resources, unclear lines of communication, and gaps in cross-sectoral planning. We have emphasized that, despite meeting the 7-1-7 detection and investigation benchmarks, MSPAS was unable to operationalize a timely field response due to these constraints.

Regarding your note about the need for comprehensive revision of the methodology and data reporting, we acknowledge the importance of ensuring clarity and rigor in these sections and have made edits throughout and in response to the other reviewer.

Reviewer #2:

This manuscript describes the application of the 7-1-7 benchmarks to an H5N1 outbreak among pelicans in Guatemala. It appropriately highlights the One Health nature of such an outbreak, given the risk of disease spillover to humans. However, there are several areas that require elaboration before this manuscript is acceptable for publication. A summary of the major areas of improvement are below, with

specific comments provided in the attached reviewer manuscript copy:

Introduction - The main issue is to delineate which specific components of the 7-1-7 benchmarks were applied here.

Sorry, we are unclear about this comment. We used the 7-1-7 benchmarks to elaborate on Guatemala's response. All components of 7-1-7 were examined in this analysis (e.g. detection, notification, and response).

Methods - The authors need to provide more information about how the interviews were conducted (particularly who conducted them and the questions asked). They also need to explain why they chose the UNICEF "Human-Centred Design 4 Health" methodology as opposed to a more traditional qualitative methodology, and they need to provide additional detail about how they used this methodology to organize and synthesize the interview information. Finally, the authors need to justify why the original interview data were not provided with the manuscript.

Thank you for your feedback on the Methods section. In response, we have now clarified who conducted the interviews and provided additional details on the types of questions asked. We have also included a more thorough description of the interview content and process in the revised manuscript. Additionally, the interview questions have been submitted as supplement 2. We also added justification about our use of the UNICEF toolkit. Specifically, we chose this approach because it is grounded in participatory design and systems thinking, which aligned closely with our goal of understanding cross-sectoral dynamics and user-centered perspectives in One Health contexts.

To address your point on how we applied this methodology, we have also expanded the data analysis section to provide a clearer explanation of how the Human-Centered Design framework guided the organization, interpretation, and synthesis of interview findings.

Finally, concerning your question about the absence of original interview data, we respectfully note that it is neither customary nor appropriate to include full interview transcripts in qualitative research manuscripts, especially when working with sensitive public health data in low-resource settings. Doing so could compromise participant confidentiality and privacy, even with anonymization.

Results - The authors need to provide high-level interviewee information in a separate table (such as role and agency, as long as the interviewees are not identifiable). The authors also need to provide a table showing how each of the three 7-1-7 steps were met and on what date, broken down by Component. This will help delineate the key rate-limiting steps, such as shipping to NVSL and inter/intra-agency coordination. Also, I'm not clear how the inability to have a meeting between MSPAS and the Izabal stakeholders falls under the poor coordination category.

Unfortunately, further information on roles within each agency cannot be provided as it would compromise the identities of the informants. We intentionally left this information out of the manuscript.

In reference to the reviewer's confusion about the need for a meeting between MSPAS and the Izabal stakeholders, we have added additional details.

In response to the reviewer's comments about the timeline, we adjusted Table 1 so it also notes the response stage of each critical activity on the timeline.

Discussion - The authors need to further describe how the enablers of outbreak detection and notification compare with the enablers in other countries (and whether those enablers similarly facilitate detection and notification).
Thanks for this comment. We have added a sentence that brought the enablers into conversation with the literature

++

Embedded comments in the PDF

Reviewer 1:

Abstract:

Comment on testing capacity: We added the word laboratory to clarify.

Introduction

Comment on evaluation tool: We clarified this issue in the comment from reviewer 2. We meant more research tools using the 717 framework.

Comment on qualitative methodology: Building on your comment, we added additional language to describe why the methodology we took was unique.

Comment on HCD toolkit: We can provide the our version of the tool as supplement in case the journal would like to publish it.

Comment on changing the AHA moments: AHA moments are surprising moments, and 3 most powerful quotes are just anything that stuck out. I think they sound distinct enough to not have to elaborate too much. If the reader wants, they can check out the UNICEF version of the tool.

Comment on changing the tool: I am not sure I understand the comment, as we did amend the tool for this particular use case.

Assessing Timeliness

Comment on effective response: the sample transferring is part of the response; however it does not constitute completion of the early response.

Emergency funding:

Comment on OIRSA funds: yes, it may turn out to be a bottleneck, but I did not want to further speculate on this matter since its beyond the scope of our argument.

Recommendations:

Comment on unique areas of insight: Your comments point to awkward language that we used. In response to this insightful comment, we amended that part to make clear what the unique insight about which we are speaking.

Limitations:

Comment on paper trail. Yes, as you point out, the retrospective nature means there was no paper trail. We amended the sentence to make this clearer.

Reviewer 2:

Introduction:

Comment on WHO JEE: The fact that such scoring is debatable, as you point out, underscores that there are shortcomings in the metric and that it could benefit from supplementation from 7-1-7 or other metrics.

Comment on early response completion: Yes, they comprise the steps of 7-1-7.

Comment on “appeared to” word: Thank you, I changed this to be more direct.

Comment on 71-17 evaluation tool suggestion: 7-1-7 is a target or metric. Tools are how we use these targets or metrics. We added some additional language to help make this clearer.

Methods

Interview comment: The comments were responded to via the emailed comments you wrote earlier. In addition, I added an appendix of the interview questions.

Comment on audio + video: It is not customary to use audio and video, so we do not think we need to add anything about that to the manuscript. We would respectfully like to point out that in qualitative work, mention of “transcription” implies that what is being written down is the language that was spoken.

Data Analysis

Comment on UNCIEF HCD: We addressed this comment in response to your emailed comments.

Results

Comment on interview table and on figure of notification pathway: These are great points to be made aware of, thank you. However, we do not think it's needed or appropriate to call out the exact occupation and background of those who are interviewed. Due to the small number of employees in each of these agencies who worked on avian influenza, delineating this could reveal their identities and leave them exposed. Second, We do not feel comfortable creating a figure on notification pathways because there is not an agreed upon notification pathway in Guatemala and anything we produce would be easily contested.

Assessing Timeliness

Comment on table for 7-1-7 steps: We addressed this comment in response to your emailed comments.

Comment on Izabal stakeholders: We changed stakeholders to community members to ensure these were just people from the community, and we did not have a clear understanding of who these people were exactly.

Emergency Funding

Comment on citation: As we find this to be common knowledge, we don't agree that it needs a citation.

Community event-based surveillance (CEBS)

Comment on demographic and linguistic composition: We added language to clarify this.

Comment on network of laboratories**:** Yes, as a private laboratory, it was one of the 4 veterinary diagnostic laboratories. We believe this is clear enough from the way it is written.

PPE:

Comment on a bag of straws: While an interesting question, we don't have further information, and We don't think elucidating this is relevant to the story.

Intra-agency and inter-agency communication and planning: We changed the leading statement, tightened language between intra and inter-agency communication, and clarified how this related to the stakeholder meeting.

Timeliness Achievements

Comment on how enablers in Guatemala could have facilitated outbreak detection in other settings: We responded to a similar comment you provided in the non-embedded, emailed comments. A sentence has been added later to the discussion to address this issue.

Recommendations

Comment on reporting bottlenecks: The reason we bring up ethical issues with focus groups/workshops in discussing outbreak proceedings is to highlight that it's a faulty method for doing so. In this situation, we did not use this method recommended by 7-1-7 literature, and this type of cross-sectoral meeting reflecting on the outbreak did not take place outside of our investigation.

Comment on focus groups: Your comment is interesting, but we think including language reflecting your question would diverge from the point of this sentence. Focus groups are not private, risk-free environments, even if anonymized. People still see, work with, and even report to others who are in an outbreak reflection focus group.

Comment on gaps: Thank you, we added language to clarify the gaps that were resolved.

Comment on interview question bias: we agree, but we don't think that is a unique challenge—all research could lead to bias no matter what approach is taken (qualitative or quantitative). Since we are not aware how the interview questions may have specifically biased the outcome, we don't agree that further information on bias and interview questions needs to be included.
