# Supplementary material for: Bottleneck and enabler evaluation of avian influenza health event — Guatemala, January-February 2023
Source: PLOS Glob Public Health. 2025 Nov 6;5(11):e0005443. doi: 10.1371/journal.pgph.0005443 (PMC12591453; doi:10.1371/journal.pgph.0005443)
Supplement: Attachment — Submitted filename: Response to Reviewers.docx [file pgph.0005443.s007.docx]

Thank you for the thoughtful and detailed review of our manuscript. We have carefully revised and responded to each point raised by the reviewers and academic editor below in red font. We have also submitted a revised manuscript in track changes, as well as an unmarked revised manuscript.

In reference to some of the high-level issues highlighted by the Academic Editor, we have made the recommended changes to the table and discussion.

**Editor comments:**

1. Please include a complete copy of PLOS’ questionnaire on inclusivity in global research in your revised manuscript. Our policy for research in this area aims to improve transparency in the reporting of research performed outside of researchers’ own country or community. The policy applies to researchers who have travelled to a different country to conduct research, research with Indigenous populations or their lands, and research on cultural artefacts. The questionnaire can also be requested at the journal’s discretion for any other submissions, even if these conditions are not met. Please find more information on the policy and a link to download a blank copy of the questionnaire here: [https://journals.plos.org/globalpublichealth/s/best-practices-in-research-reporting](https://nam11.safelinks.protection.outlook.com/?url=https%3A%2F%2Fjournals.plos.org%2Fglobalpublichealth%2Fs%2Fbest-practices-in-research-reporting&data=05%7C02%7Cpsuchde%40emory.edu%7C653e553cf0274171fe7308ddcb2f4c6d%7Ce004fb9cb0a4424fbcd0322606d5df38%7C0%7C0%7C638890126479015725%7CUnknown%7CTWFpbGZsb3d8eyJFbXB0eU1hcGkiOnRydWUsIlYiOiIwLjAuMDAwMCIsIlAiOiJXaW4zMiIsIkFOIjoiTWFpbCIsIldUIjoyfQ%3D%3D%7C0%7C%7C%7C&sdata=jER1UkDWMmaJ6KWtihCsaNfhFrAl1LP5PA7qPAO4sBs%3D&reserved=0). Please upload a completed version of your questionnaire as Supporting Information when you resubmit your manuscript.

- Done

2. We do not publish any copyright or trademark symbols that usually accompany proprietary names, eg (R), (C), or TM (e.g. next to drug or reagent names). Please remove all instances of trademark/copyright symbols throughout the text, including ® on page 15.

- Done

3. We note that your Data Availability Statement is currently as follows: [All relevant data are within the paper.]

   Please confirm at this time whether or not your submission contains all raw data required to replicate the results of your study. Authors must share the “minimal data set” for their submission. PLOS defines the minimal data set to consist of the data required to replicate all study findings reported in the article, as well as related metadata and methods ([https://journals.plos.org/plosone/s/data-availability#loc-minimal-data-set-definition](https://nam11.safelinks.protection.outlook.com/?url=https%3A%2F%2Fjournals.plos.org%2Fplosone%2Fs%2Fdata-availability%23loc-minimal-data-set-definition&data=05%7C02%7Cpsuchde%40emory.edu%7C653e553cf0274171fe7308ddcb2f4c6d%7Ce004fb9cb0a4424fbcd0322606d5df38%7C0%7C0%7C638890126479031362%7CUnknown%7CTWFpbGZsb3d8eyJFbXB0eU1hcGkiOnRydWUsIlYiOiIwLjAuMDAwMCIsIlAiOiJXaW4zMiIsIkFOIjoiTWFpbCIsIldUIjoyfQ%3D%3D%7C0%7C%7C%7C&sdata=ZTwci4P1sq15klUaYFMaHPaACseMAt69ZNxjvpMO7Ss%3D&reserved=0)).

   For example, authors should submit the following data:
   - The values behind the means, standard deviations and other measures reported;
   - The values used to build graphs;
   - The points extracted from images for analysis.
   Authors do not need to submit their entire data set if only a portion of the data was used in the reported study.
   If your submission does not contain these data, please either upload them as Supporting Information files or deposit them to a stable, public repository and provide us with the relevant URLs, DOIs, or accession numbers. For a list of recommended repositories, please see [https://journals.plos.org/plosone/s/recommended-repositories](https://nam11.safelinks.protection.outlook.com/?url=https%3A%2F%2Fjournals.plos.org%2Fplosone%2Fs%2Frecommended-repositories&data=05%7C02%7Cpsuchde%40emory.edu%7C653e553cf0274171fe7308ddcb2f4c6d%7Ce004fb9cb0a4424fbcd0322606d5df38%7C0%7C0%7C638890126479047350%7CUnknown%7CTWFpbGZsb3d8eyJFbXB0eU1hcGkiOnRydWUsIlYiOiIwLjAuMDAwMCIsIlAiOiJXaW4zMiIsIkFOIjoiTWFpbCIsIldUIjoyfQ%3D%3D%7C0%7C%7C%7C&sdata=3WmPLi1Q9%2BFCAjsGLo8L8NFySENdu5LPEhgRjGgmi1E%3D&reserved=0).

   If there are ethical or legal restrictions on sharing a de-identified data set, please explain them in detail (e.g., data contain potentially sensitive information, data are owned by a third-party organization, etc.) and who has imposed them (e.g., an ethics committee). Please also provide contact information for a data access committee, ethics committee, or other institutional body to which data requests may be sent. If data are owned by a third party, please indicate how others may request data access.

- **Confirmed**

**Reviewer #1:**

The manuscript is well written and presents a valuable and timely contribution to One Health area. The content is easy to comprehend and the data is relevant. However, the manuscript would benefit from a comprehensive revision, particularly with methodology and data reporting. It would also be great to see further more elaboration on causes/reasons of no 'on-the-ground' action by MSPAS (shared paragraph below). This no-action delayed the effective response even when they managed to adhere to 7-1-7 for detection and investigation initiation phases with limited resource constraint.

"However, internal agency communication and planning in MSPAS appeared to be even more key in delaying the response. Once MSPAS received the notification, they did not take any on-the-ground action until at least March 1st. It took MSPAS several weeks to find the personnel, equipment, and funding. Thus, even with the delay in notification from MAGA, there appeared to be unclear lines of communication within MSPAS to initiate a cross-sectoral response that would protect humans against potential disease spillover."

We thank you for these comments and constructive feedback. We appreciate your recognition of the manuscript's contribution to the One Health field and your comments on its clarity and relevance. In response to your suggestion regarding elaboration on the lack of 'on-the-ground' action by MSPAS, we have revised the paragraph to more clearly articulate that the delay stemmed from internal challenges, including limited resources, unclear lines of communication, and gaps in cross-sectoral planning. We have emphasized that, despite meeting the 7-1-7 detection and investigation benchmarks, MSPAS was unable to operationalize a timely field response due to these constraints.

Regarding your note about the need for comprehensive revision of the methodology and data reporting, we acknowledge the importance of ensuring clarity and rigor in these sections and have made edits throughout and in response to the other reviewer.

**Reviewer #2**:

This manuscript describes the application of the 7-1-7 benchmarks to an H5N1 outbreak among pelicans in Guatemala. It appropriately highlights the One Health nature of such an outbreak, given the risk of disease spillover to humans. However, there are several areas that require elaboration before this manuscript is acceptable for publication. A summary of the major areas of improvement are below, with specific comments provided in the attached reviewer manuscript copy:

Introduction - The main issue is to delineate which specific components of the 7-1-7 benchmarks were applied here.

Sorry, we are unclear about this comment. We used the 7-1-7 benchmarks to elaborate on Guatemala’s response. All components of 7-1-7 were examined in this analysis (e.g. detection, notification, and response).

Methods - The authors need to provide more information about how the interviews were conducted (particularly who conducted them and the questions asked). They also need to explain why they chose the UNICEF "Human-Centred Design 4 Health" methodology as opposed to a more traditional qualitative methodology, and they need to provide additional detail about how they used this methodology to organize and synthesize the interview information. Finally, the authors need to justify why the original interview data were not provided with the manuscript.

Thank you for your feedback on the Methods section. In response, we have now clarified who conducted the interviews and provided additional details on the types of questions asked. We have also included a more thorough description of the interview content and process in the revised manuscript. Additionally, the interview questions have been submitted as supplement 2. We also added justification about our use of the UNICEF toolkit. Specifically, we chose this approach because it is grounded in participatory design and systems thinking, which aligned closely with our goal of understanding cross-sectoral dynamics and user-centered perspectives in One Health contexts.

To address your point on how we applied this methodology, we have also expanded the data analysis section to provide a clearer explanation of how the Human-Centered Design framework guided the organization, interpretation, and synthesis of interview findings.

Finally, concerning your question about the absence of original interview data, we respectfully note that it is neither customary nor appropriate to include full interview transcripts in qualitative research manuscripts, especially when working with sensitive public health data in low-resource settings. Doing so could compromise participant confidentiality and privacy, even with anonymization.

Results - The authors need to provide high-level interviewee information in a separate table (such as role and agency, as long as the interviewees are not identifiable). The authors also need to provide a table showing how each of the three 7-1-7 steps were met and on what date, broken down by Component. This will help delineate the key rate-limiting steps, such as shipping to NVSL and inter/intra-agency coordination. Also, I'm not clear how the inability to have a meeting between MSPAS and the Izabal stakeholders falls under the poor coordination category.

Unfortunately, further information on roles within each agency cannot be provided as it would compromise the identities of the informants. We intentionally left this information out of the manuscript.

In reference to the reviewer’s confusion about the need for a meeting between MSPAS and the Izabal stakeholders, we have added additional details.

In response to the reviewer’s comments about the timeline, we adjusted Table 1 so it also notes the response stage of each critical activity on the timeline.

Discussion - The authors need to further describe how the enablers of outbreak detection and notification compare with the enablers in other countries (and whether those enablers similarly facilitate detection and notification).
Thanks for this comment. We have added a sentence that brought the enablers into conversation with the literature

+++++++++++++++++++++++++++++++++++++++++++++++++++++++++++++++++++++++++

**Embedded comments in the PDF**

Reviewer 1:

**Abstract:**

Comment on testing capacity: We added the word laboratory to clarify.

**Introduction**

Comment on evaluation tool: We clarified this issue in the comment from reviewer 2. We meant more research tools using the 717 framework.

Comment on qualitative methodology: Building on your comment, we added additional language to describe why the methodology we took was unique.

Comment on HCD toolkit: We can provide the our version of the tool as supplement in case the journal would like to publish it.

Comment on changing the AHA moments: AHA moments are surprising moments, and 3 most powerful quotes are just anything that stuck out. I think they sound distinct enough to not have to elaborate too much. If the reader wants, they can check out the UNICEF version of the tool.

Comment on changing the tool: I am not sure I understand the comment, as we did amend the tool for this particular use case.

**Assessing Timeliness**

Comment on effective response: the sample transferring is part of the response; however it does not constitute completion of the early response.

**Emergency funding:**

Comment on OIRSA funds: yes, it may turn out to be a bottleneck, but I did not want to further speculate on this matter since its beyond the scope of our argument.

**Recommendations:**

Comment on unique areas of insight: Your comments point to awkward language that we used. In response to this insightful comment, we amended that part to make clear what the unique insight about which we are speaking.

**Limitations:**

Comment on paper trail. Yes, as you point out, the retrospective nature means there was no paper trail. We amended the sentence to make this clearer.

Reviewer 2:

**Introduction:**

Comment on WHO JEE: The fact that such scoring is debatable, as you point out, underscores that there are shortcomings in the metric and that it could benefit from supplementation from 7-1-7 or other metrics.

Comment on early response completion: Yes, they comprise the steps of 7-1-7.

Comment on “appeared to” word: Thank you, I changed this to be more direct.

Comment on 71-17 evaluation tool suggestion: 7-1-7 is a target or metric. Tools are how we use these targets or metrics. We added some additional language to help make this clearer.

**Methods**

Interview comment: The comments were responded to via the emailed comments you wrote earlier. In addition, I added an appendix of the interview questions.

Comment on audio + video: It is not customary to use audio and video, so we do not think we need to add anything about that to the manuscript. We would respectfully like to point out that in qualitative work, mention of “transcription” implies that what is being written down is the language that was spoken.

**Data Analysis**

Comment on UNCIEF HCD: We addressed this comment in response to your emailed comments.

**Results**

Comment on interview table and on figure of notification pathway: These are great points to be made aware of, thank you. However, we do not think it’s needed or appropriate to call out the exact occupation and background of those who are interviewed. Due to the small number of employees in each of these agencies who worked on avian influenza, delineating this could reveal their identities and leave them exposed. Second, We do not feel comfortable creating a figure on notification pathways because there is not an agreed upon notification pathway in Guatemala and anything we produce would be easily contested.

**Assessing Timeliness**

Comment on table for 7-1-7 steps: We addressed this comment in response to your emailed comments.

Comment on Izabal stakeholders: We changed stakeholders to community members to ensure these were just people from the community, and we did not have a clear understanding of who these people were exactly.

**Emergency Funding**

Comment on citation: As we find this to be common knowledge, we don’t agree that it needs a citation.

**Community event-based surveillance (CEBS)**

Comment on demographic and linguistic composition: We added language to clarify this.

Comment on network of laboratories**:** Yes, as a private laboratory, it was one of the 4 veterinary diagnostic laboratories. We believe this is clear enough from the way it is written.

**PPE:**

Comment on a bag of straws: While an interesting question, we don’t have further information, and We don’t think elucidating this is relevant to the story.

**Intra-agency and inter-agency communication and planning:** We changed the leading statement, tightened language between intra and inter-agency communication, and clarified how this related to the stakeholder meeting.

**Timeliness Achievements**

Comment on how enablers in Guatemala could have facilitated outbreak detection in other settings: We responded to a similar comment you provided in the non-embedded, emailed comments. A sentence has been added later to the discussion to address this issue.

**Recommendations**

Comment on reporting bottlenecks: The reason we bring up ethical issues with focus groups/workshops in discussing outbreak proceedings is to highlight that it’s a faulty method for doing so. In this situation, we did not use this method recommended by 7-1-7 literature, and this type of cross-sectoral meeting reflecting on the outbreak did not take place outside of our investigation.

Comment on focus groups: Your comment is interesting, but we think including language reflecting your question would diverge from the point of this sentence. Focus groups are not private, risk-free environments, even if anonymized. People still see, work with, and even report to others who are in an outbreak reflection focus group.

Comment on gaps: Thank you, we added language to clarify the gaps that were resolved.

Comment on interview question bias: we agree, but we don’t think that is a unique challenge—all research could lead to bias no matter what approach is taken (qualitative or quantitative). Since we are not aware how the interview questions may have specifically biased the outcome, we don’t agree that further information on bias and interview questions needs to be included.
