# Supplementary material for: Bottleneck and enabler evaluation of avian influenza health event — Guatemala, January-February 2023
Source: PLOS Glob Public Health. 2025 Nov 6;5(11):e0005443. doi: 10.1371/journal.pgph.0005443 (PMC12591453; doi:10.1371/journal.pgph.0005443)
Supplement: Attachment — Submitted filename: Response to Reviewers 2_2SEP25.docx [file pgph.0005443.s008.docx]

Thank you for the additional review of our manuscript. We have carefully revised and responded to the remaining points raised by the editor and reviewers below in red font. We have also submitted a revised manuscript in track changes, as well as an unmarked revised manuscript.

**Editor comments:**

1. Please include a complete copy of PLOS’ questionnaire on inclusivity in global research in your revised manuscript. Our policy for research in this area aims to improve transparency in the reporting of research performed outside of researchers’ own country or community. The policy applies to researchers who have travelled to a different country to conduct research, research with Indigenous populations or their lands, and research on cultural artefacts. The questionnaire can also be requested at the journal’s discretion for any other submissions, even if these conditions are not met. Please find more information on the policy and a link to download a blank copy of the questionnaire here: [https://journals.plos.org/globalpublichealth/s/best-practices-in-research-reporting](https://nam11.safelinks.protection.outlook.com/?url=https%3A%2F%2Fjournals.plos.org%2Fglobalpublichealth%2Fs%2Fbest-practices-in-research-reporting&data=05%7C02%7Cpsuchde%40emory.edu%7C653e553cf0274171fe7308ddcb2f4c6d%7Ce004fb9cb0a4424fbcd0322606d5df38%7C0%7C0%7C638890126479015725%7CUnknown%7CTWFpbGZsb3d8eyJFbXB0eU1hcGkiOnRydWUsIlYiOiIwLjAuMDAwMCIsIlAiOiJXaW4zMiIsIkFOIjoiTWFpbCIsIldUIjoyfQ%3D%3D%7C0%7C%7C%7C&sdata=jER1UkDWMmaJ6KWtihCsaNfhFrAl1LP5PA7qPAO4sBs%3D&reserved=0). Please upload a completed version of your questionnaire as Supporting Information when you resubmit your manuscript.

- Done

2. If the reviewer comments include a recommendation to cite specific previously published works, please review and evaluate these publications to determine whether they are relevant and should be cited. There is no requirement to cite these works unless the editor has indicated otherwise.
3. Please review your reference list to ensure that it is complete and correct. If you have cited papers that have been retracted, please include the rationale for doing so in the manuscript text, or remove these references and replace them with relevant current references. Any changes to the reference list should be mentioned in the rebuttal letter that accompanies your revised manuscript. If you need to cite a retracted article, indicate the article’s retracted status in the References list and also include a citation and full reference for the retraction notice.

**Reviewer #1:**

Thanks for all the updates. I have few more requests on methodology section.

- Responses to suggested edits in the pdf have been made in the revised manuscript.
- We felt it was outside our scope to respond to the reviewer’s suggestion to add a diagram of the qualitative methodology we utilized.
- Response to questions in Methods section:
  - The three quotes presented in the results were not arbitrarily chosen from the initial five quotes identified per interview. Rather, they emerged from the second stage of analysis, when the “Record Field Research” worksheets were grouped into subgroups and synthesized. The final three quotes presented in the manuscript reflect a deliberate narrowing process embedded in the toolkit’s analytic framework, rather than subjective selection by the authors. We have added the following sentence to clarify: “From the five meaningful quotes identified per interview, the three most representative and compelling quotes were selected during the subgroup-level synthesis stage, following the UNICEF toolkit framework but extended in our study through systematic comparison across subgroups.”
  - This study followed UNICEF’s overall analysis process but differed in how the synthesis stage was applied. While UNICEF treats completion of the “Synthesis Tool” as the final step, our study extended the analysis by systematically comparing subgroup syntheses (national MAGA, local MAGA, national MSPAS, local MSPAS). This additional layer allowed us to identify cross-cutting themes and divergences between the human and animal health sectors, an approach not emphasized in the UNICEF toolkit.
  - We see “AHA! moments” and “powerful quotes” as related but distinct. “AHA! moments” in the original UNICEF toolkit are designed to capture points of surprise that might reveal hidden motivations, behaviors, or opportunities for intervention. By contrast, our adapted “powerful quotes” category aimed to capture particularly striking, memorable, or representative statements that distilled key perspectives, whether or not they were surprising. While there may be overlap (an AHA! moment could also be a powerful quote), not all powerful quotes are surprising, and not all surprising moments are necessarily the best illustrations of group perspectives. Separating the two categories allowed us to highlight both unexpected insights and representative voices. We have modified the last paragraph of the Data analysis section.

**Reviewer #2:**

1. The word "fisherman" in the Introduction and Results section should be plural ("fishermen")

- Thank you. Done.

2. In the Results section under Assessing Timeliness, you should make it clear upfront that H5 was confirmed in Guatemala City, but the sample needed to go to NVSL to confirm H5N1. I also recommend noting when MSPAS received the notification from MAGA. Also, I think you can delete column 3 in Table 1, which appears redundant.

- We appreciate these comments and have made these changes.

3. In the Discussion section under Recommendations, it's not clear what's the second obstacle in carrying out 7-1-7 assessments - I recommend making this more explicit.

- Thanks for this suggestion. We have edited this paragraph.

4. Finally, although sharing interview transcripts with manuscripts is uncommon, I would like to gently push back against the notion that sharing such qualitative research data in general is inappropriate. In fact, it is increasingly expected that interview or focus group transcripts be shared (typically with a data repository - see <https://www.pnas.org/doi/10.1073/pnas.2206981120>). Choosing not to share such data for fears of interviewee re-identification should be the exception, not the norm, and there are tools available to help mitigate these concerns (see previous article). In the future, I'd encourage the authors to consider whether interview data sharing is a realistic possibility rather than discounting the possibility outright, as appears to have been the case here. For now, I believe it is appropriate to note in the Data Sharing section that raw interview data are unavailable because of privacy concerns.

- Thank you for this feedback which we will share with our partners for consideration in future studies.
